# Significant Impact of a Daytime Halogen Oxidant on Coastal Air Quality

**DOI:** 10.1021/acs.est.4c08360

**Published:** 2025-01-24

**Authors:** Jianing Dai, Tao Wang, Hengqing Shen, Men Xia, Weihang Sun, Guy P. Brasseur

**Affiliations:** † Department of Civil and Environmental Engineering, The Hong Kong Polytechnic University, Hong Kong SAR 999077, China; ‡ Environmental Modelling Group, Max Planck Institute for Meteorology, Hamburg 20146, Germany; § NSF-National Center for Atmospheric Research, Boulder, Colorado 80307, United States; ∥ Institute for Atmospheric and Earth System Research/Physics, Faculty of Science, University of Helsinki, Helsinki 00014, Finland; ⊥ Aerosol and Haze Laboratory, Beijing Advanced Innovation Center for Soft Matter Science and Engineering, Beijing University of Chemical Technology, Beijing 100029, China

**Keywords:** molecular chlorine, air pollution, atmospheric oxidation, WRF-Chem

## Abstract

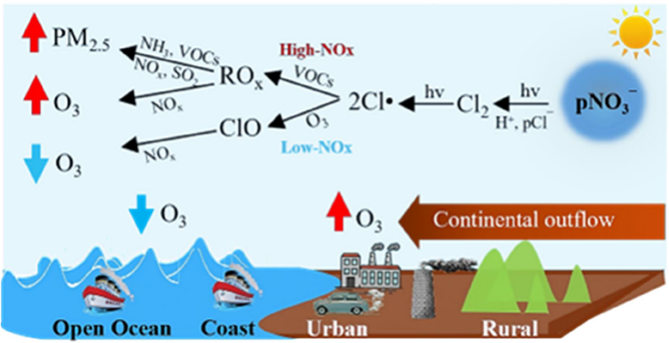

Chlorine radicals (Cl^·^) are highly reactive and affect the fate of air pollutants. Several field studies in China have revealed elevated levels of daytime molecular chlorine (Cl_2_), which, upon photolysis, release substantial amounts of Cl^·^ but are poorly represented in current chemical transport models. Here, we implemented a parametrization for the formation of daytime Cl_2_ through the photodissociation of particulate nitrate in acidic environments into a regional model and assessed its impact on coastal air quality during autumn in South China. The model could reproduce over 70% of the high Cl_2_ level measured at a coastal site, revealing a discernible presence of Cl_2_ and released Cl^·^ in coastal and adjacent areas. Abundant Cl_2_ alters the oxidative capacity of the atmosphere, consequently increasing O_3_ (6–12%) and PM_2.5_ (10–16%) concentrations in high-NO_*x*_ areas and reducing O_3_ (3%) concentration in low-NO_*x*_ areas. Accounting for chlorine chemistry shifts the O_3_ – precursor relationships from VOC limited to mixed or NO_*x*_ -limited regimes, enhancing the benefits of NO_*x*_ emission reduction in mitigating O_3_ pollution. Our findings suggest that tightening emission control for two acidic pollutants, NO_*x*_ and SO_2_, would alleviate reactive Cl^·^ production and its adverse impact on air quality.

## Abstract 2

Elevated levels of molecular chlorine significantly affect the production of ozone and secondary aerosol in polluted coastal environments, with implications for the ozone control strategy and assessment of air pollution.

## 1 Introduction

Chlorine radicals (Cl^·^) are potent oxidants and influence the abundances of climate-relevant and air-quality-relevant trace gases.^1−5^ Cl^·^ has been known to destroy ozone (O_3_) through catalytic cycles (R1), altering the oxidation capacity of the atmosphere.^6^ In the lower troposphere, the presence of volatile organic compounds (VOCs) triggers the Cl^·^-initiated oxidation of VOCs (R2), which enhances the levels of conventional radicals (OH^·^, HO_2_^·^, and RO_2_^·^) and promotes the formation of O_3_ (R3–R7) and secondary aerosols R8.^7^ In addition, Cl^·^ can react with greenhouse gases, such as O_3_ and methane (CH_4_) R9, which alters the global radiative forcing and has climate implications.^8,9^
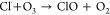
R1
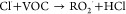
R2
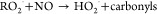
R3
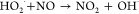
R4
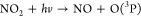
R5
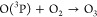
R6
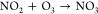
R7
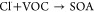
R8
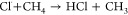
R9

Cl^·^ is primarily produced by the activation of reactive inorganic chlorine species.^10^ Among the chlorine species, nitryl chloride (ClNO_2_) has been recognized as a major Cl^·^ precursor in the polluted troposphere R10.^11−16^ ClNO_2_ is produced mostly at night via heterogeneous reactions of dinitrogen pentoxide (N_2_O_5_) with chlorine-containing aerosols,^11^ serving as a nocturnal reservoir of chlorine and nitrogen oxides (NO_*x*_). After sunrise, the photolysis of ClNO_2_ rapidly produces Cl^·^ in the early morning.^15,16^ Molecular chlorine (Cl_2_) is another potentially important precursor for Cl^·^ and has been observed in locations such as the Arctic surface,^17,18^ the marine and coastal areas,^19−23^ and continental sites R11.^2,15,23−25^ Cl_2_ has been found to typically peak during nighttime, partially explained by the uptake of ClNO_2_ and N_2_O_5_.^15,21^ Recently, elevated high levels of daytime Cl_2_ have also been measured at an inland rural site in North China (up to 450 pptv) and at polluted coastal sites over South (up to 998 pptv) and East China (up to 1100 pptv).^22,23,25^ The presence of daytime Cl_2_ is significant as it reveals a strong source of Cl· given that the photolytic lifetime of Cl_2_ is short (e.g., ∼7 min at autumn noontime in South China^22^) and that the photodissociation of Cl_2_ produces two Cl·, which can profoundly impact atmospheric photochemistry and oxidation capacity.
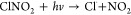
R10
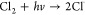
R11

Several chemical mechanisms have been proposed to explain daytime Cl_2_ production, including autocatalytic halogen activation,^26,27^ the aqueous-phase reaction of OH^·^ on acidic chloride-containing aerosol,^28^ O_3_ uptake by aerosol,^29^ and aerosol iron (Fe) photochemistry.^30,31^ While the first two mechanisms have been incorporated into box models, these could not account for the high level of daytime Cl_2_ measured at a polluted coastal site in South China.^22^ Based on regional models, the autocatalytic halogen activation has also been proven to play only a limited role in the daytime Cl_2_ observed in the eastern United States^35^ and in North and East China.^32−34^ Studies have also incorporated O_3_ uptake over chorine-containing aerosols into box models and regional chemical transport models.^32−34^ However, these models could only reproduce the observed Cl_2_ concentrations with large O_3_ uptake coefficients, which are not supported by available laboratory measurements.^34^ A recent study on Fe(III)-induced photolytic Cl_2_ formation in the GEOS-Chem model explained one-third of the daytime Cl_2_ measured at a polluted rural site in North China,^36^ with a large fraction remaining unaccounted for.

Based on field and laboratory results, Peng et al.^22^ demonstrated that chloride activation via the photolysis of particulate nitrate could generate Cl_2_ under acidic conditions (pH < 3.3), and their box model calculations suggest that this photolytic source could explain a large fraction of the production rates of Cl_2_ at that site. The calculations with a photochemical box model constrained by the observations data indicated that the daytime Cl_2_ considerably affects local oxidation capacity, increasing OH^·^, HO_2_^·^, and RO_2_^·^ by 4, 17, and 27%, respectively. However, the geographical extent of such an impact remains unquantified due to the lack of representation of this daytime source in current chemical transport models. Moreover, it is unclear how this daytime oxidant would affect current ozone control strategy.

In this study, we developed a parametrization for daytime Cl_2_ production based on the laboratory and field measurements of Peng et al.^22^ and implemented it in a regional chemical transport model (WRF-Chem) to evaluate the impact of daytime Cl_2_ and other reactive chlorine species on the secondary pollutants during autumn of 2018. We show that the improved model can reproduce the variations and magnitudes of the observed Cl_2_ and that the daytime Cl_2_ substantially enhanced the production of radicals, O_3_, and secondary aerosols in the coastal areas of South China. We also demonstrate that reactive halogen chemistry can reshape the O_3_-precursor relationship and designation of O_3_-formation regimes. Finally, we show that reactive chlorine chemistry can also improve the efficiency of further reduction in NO_*x*_ and sulfur dioxide (SO_2_) emissions in mitigating O_3_ pollution. This study yields new insights into and calls for more research on under-explored sources and the impact of reactive halogens in polluted environments.

## 2 Materials and Methods

### 2.1 Model Configuration and Inputs

The WRF-Chem model (version 4.1.2),^37^ coupled with the MOZART gas-phase chemistry mechanism^38^ and the MOSAIC aerosol module,^39^ was used to simulate the meteorological fields as well as the transport and the chemical and physical transformations of trace gases and aerosols. The default WRF-Chem model did not comprehensively consider chlorine-related reactions with MOZART-MOSAIC mechanism. Our previous studies^41^ have added into the model the reactions for the formation and the photolysis of several chlorine species, including hydrogen chloride (HCl), hypochlorous acid (HOCl), ClNO_2_, chlorine nitrate (ClNO_3_), and chlorine monoxide (ClO), as well as the oxidation reactions between Cl^·^ and VOCs (see R2–R46 in Table S1). Moreover, multiple nitrous acid (HONO) sources, including gas-phase reactions between NO_*x*_ and HO_*x*_, heterogeneous reactions of NO_2_ on the particle and ground, photolysis of particulate nitrate, and direct emission from vehicles and soils, have been included in the model (see Table S2).^61,62^ The HO_2_^·^ and NO_3_^·^ uptake on aerosol surfaces have also been previously incorporated into the WRF-Chem model.^40^

Several data sets were used as chemical and meteorological inputs to initiate and drive the WRF-Chem model. Data set ds083.2 provided by the National Centers for Environmental Prediction (NCEP; https://rda.ucar.edu/datasets/) was used as the meteorological input. Data sets ds351.0 and ds461.0 were used for data assimilation of meteorological simulation in the WRF model. The output from the CAM-Chem model, a global chemistry and climate model, were used as chemical initial and boundary conditions. Three sets of anthropogenic emission inventories (EI) were adopted for the routine air pollutants [NO_*x*_, SO_2_, carbon monoxide (CO), VOCs, ammonia (NH_3_), fine particulate matter (PM_2.5_), coarse particulate matter (PM_10_)], including the EI developed by the Hong Kong Environmental Protection Department (HKEPD; https://cd.epic.epd.gov.hk/) for Hong Kong in 2017, the EI developed by the South China University of Technology for the PRD region in 2017,^42^ and the Multi-resolution Emission Inventory for China (MEIC; http://www.meicmodel.org/) developed by Tsinghua University for mainland China, excluding the PRD, in 2018.

**Figure 1:**
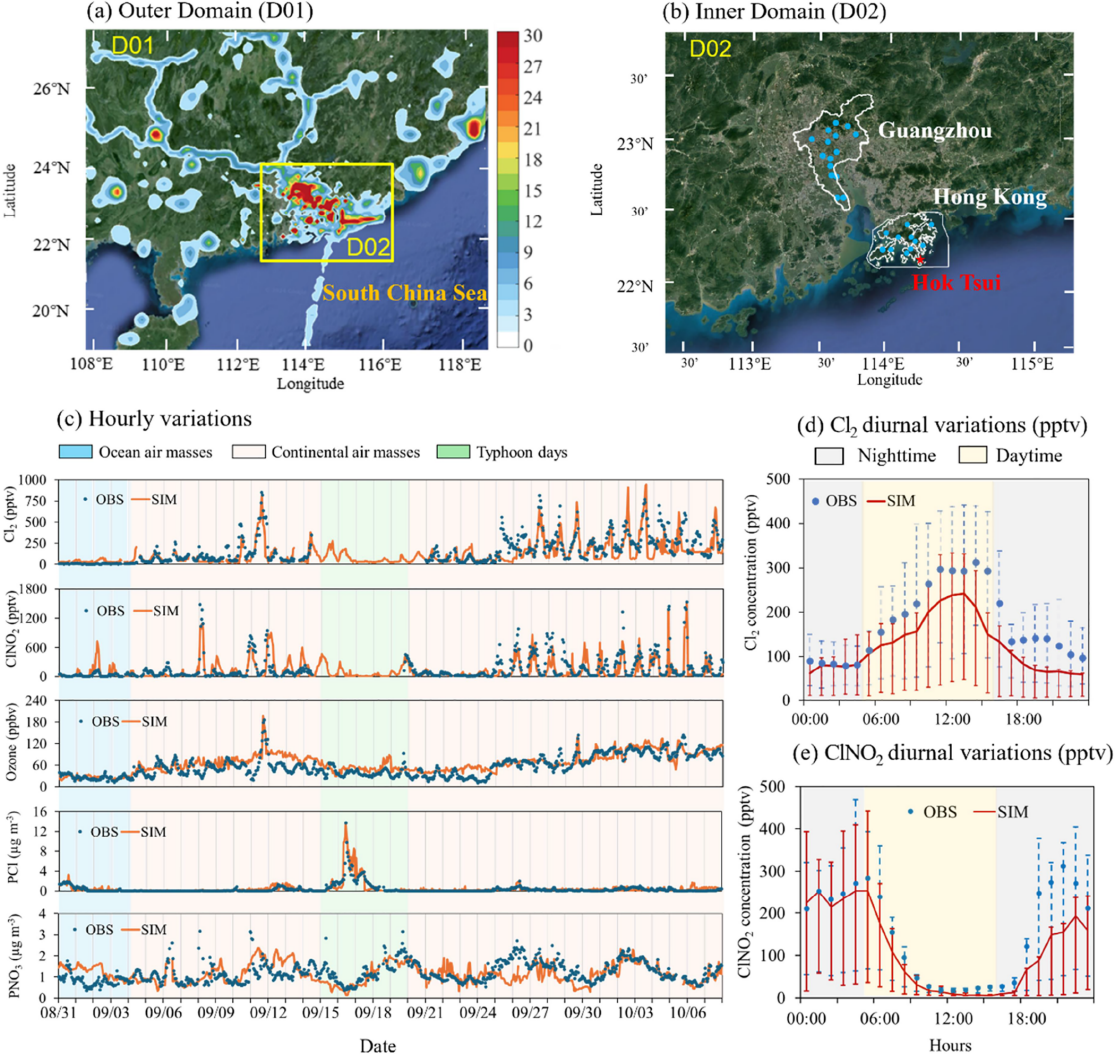
Model validations for Cl_2_, ClNO_2_, and Cl_2_-related species. (a) Outer model domain in South China (D01) with anthropogenic NO_*x*_ emissions flux (unit: molecule km^–2^ h^–1^). (b) Inner domain (D02) and locations of monitoring sites. The red star represents the location of the sampling site at Cape D’Aguilar (also called Hok Tsui); the blue dots inside the boundary lines (in white) represent the regulatory monitoring sites in Hong Kong and Guangzhou. (c) Hourly variations in simulated (in CL case) and observed mixing ratios of Cl_2_ (unit: pptv), ClNO_2_ (unit: pptv), and ozone (O_3_, unit: ppbv) and concentrations of fine particulate chloride (*p*Cl^–^, unit: μg m^–3^) and fine particulate nitrate (*p*NO_3_^–^, unit: μg m^–3^) at Cape D’Aguilar from August 31 to October 7, 2018. The periods shaded in light blue, red, and green represent marine inflow, continental outflow, and typhoon days, respectively, which are determined based on 72- hour backward trajectories.^22^ (d and e) Campaign-averaged diurnal variations in observed and simulated (in CL case) mixing ratios of (d) Cl_2_ and (e) ClNO_2_ at the Cape D’Aguilar site, with error bars representing 25th and 75th percentiles. Background maps in panels (a and b) are sourced from Google Earth (https://earth.google.com/).

Regarding chlorine emissions, the natural emissions of particulate chloride (Cl^–^) were estimated online using the mechanism described by Dai et al.,^3^ with the natural emissions of HCl through the partitioning processes of chloride. Anthropogenic chlorine emissions, including HCl and fine particulate Cl^–^, from the burning activities of coal, biomass, and municipal solid waste, were sourced from Fu et al.,^43^ with the spatial distribution and the hourly and monthly variations of the emission shown in Figure S1. The doubled-nested domains, covering the South China and Pearl River Delta (PRD) regions (Figure 1a,b), were used with horizontal resolutions of 9km and 3km, respectively. More details on the model configuration can be found in the Supporting Information (SI; Table S3).^40^

### 2.2 Updated Secondary Production of Cl_2_

We previously updated the photolysis of Cl_2_ (R1 in Table S2) and two gas-phase reactions for Cl_2_ production in the WRF-Chem model (R20, R24 in Table S2).^37^ However, the contribution of these reactions to Cl_2_ production is limited due to the low reaction rates.^6^ Thus, we further updated the Cl_2_ production and other chlorine-related reactions in the model, as described in the following subsections.

#### 2.2.1 Parametrization of Cl_2_ Production via Nitrate Photolysis

Recent laboratory studies have demonstrated the hydroxyl radical (OH^·^) or oxygen atom (O(^3^P)) from the nitrate photolysis and subsequent oxidation of chloride in solution could produce substantial amount Cl_2_ production,^22,44,45^ with the production rate of Cl_2_ (*P*(Cl_2_)) being positively linked to the values of acidity and surface area of the solution.^22^ Field measurements show a strong positive correlation between *P*(Cl_2)_ and the product of solar radiation, ambient concentration of nitrate, and aerosol surface area. The *P*(Cl_2_) did not appear to depend on particulate chlorine.^22^ Based on these experimental results, we derived a parametrization based on the linear fitting of field campaign measurements at Cape D’Aguilar for the production of daytime Cl_2_ from nitrate photolysis as follows:
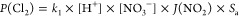
E1where [H^+^] and [NO_3_^–^] (unit: mol L^–1^) represent the aqueous-phase concentrations of hydrogen ions and nitrate ions in PM_2.5_ aerosol, which are calculated using the ISORROPIA-II (see SI Text S1 for model configuration and input); *J*(NO_2_) (unit: s^–1^) and *S*_a_ (unit: μm^2^ cm^–3^) are the photolysis rate of NO_2_ and the aerosol surface area density, respectively; and *k*_1_ denotes an empirical prefactor and has no explicit physical meaning. The daytime production rate of Cl_2_ [*P*(Cl_2_); unit: pptv s^–1^] is assumed to be the same as the consumption rate when photodissociation is the primary loss pathway (*P*(Cl_2_) = *J*(Cl_2_) × [Cl_2_]). Based on the linear fitting of field campaign measurements at Cape D’Aguilar, the slope (*k*_1_) in the parametrization was determined to be 28.91 (Figure S2a).

The correlation coefficient (*R*^2^) for the linear fitting of *P*(Cl_2_) was moderate (0.57; Figure S2a), which could be due to uncertainties in estimating the aqueous-phase [H^+^] by using ISORROPIA-II and contributions from other factors not considered in the parametrization. Our parametrization did not include the chloride concentration, because the inclusion of fine particulate chlorides in the parametrization of *P*(Cl_2_) would weaken the correlation (Figure S2b). Our previous studies also revealed that fine particulate chlorides are abundantly sourced from anthropogenic activities in South China.^46,47^ These results suggest that chloride should not be a limiting factor for Cl_2_ production in the study region. Despite uncertainties in the parametrization, our model could reproduce ∼70% of the daytime Cl_2_ concentration at Cape D’Aguilar (see Section 3.1). Moreover, our model with the aforementioned parametrization could reproduce (81%) daytime Cl_2_ concentrations at Cape D’Aguilar recently measured in 2023 (Figure S3), indicating the robustness of our parametrization in reproducing the daytime Cl_2_ concentration at the site.

We note that although nitrate photolysis leads to coproduction of Cl_2_ and HONO (e.g., Peng et al.^22^), the parametrizations for the production rates take different forms. The commonly used (and adopted in our study) HONO production rate is proportional to solar radiation and aerosol nitrate concentrations (*p*NO_3_^–^ → 0.67HONO + 0.33NO_2,_*J*_*p*NO3_ = (8.3 × 10^–5^/7 × 10^–7^) × *J*_HNO3_), whereas the Cl_2_ production rate is proportional to solar radiation, nitrate and hydrogen ion concentrations in aerosol water and aerosol surface density. Despite different formulations, the Cl_2_ and HONO productions from nitrate photolysis and their interactions have been considered in our model.

#### 2.2.2 Parametrization of Cl_2_ Production via N_2_O_5_ Uptake

Another chemical mechanism of Cl_2_ production, which occurs predominantly at night, was also considered in this study. Xia et al.^15^ found the measured Cl_2_ and ClNO_2_ significantly correlated on most nights at a subrural site in East China and considered this as evidence of Cl_2_ being a coproduct, along with ClNO_2_, of N_2_O_5_ uptake on chlorine-containing acidic aerosols. Accordingly, Xia et al.^15^ developed a parametrization of Cl_2_ yield [φ(Cl_2_)] based on the derived N_2_O_5_ uptake and ClNO_2_ yield, which is expressed as follows E2:
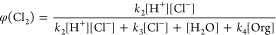
E2where [H^+^], [Cl^–^], and [Org] (unit: mol L^–1^) represent the aqueous-phase concentrations of aerosol H^+^, fine particulate Cl^–^, and organic aerosols, respectively; *k*_2_, *k*_3_, and *k*_4_ are calculated to be 19.38, 483, and 2.06, respectively. Due to the lack of organic aerosol data during our field campaign, we did not replicate this method with our data in Hong Kong, but adopted E2 in the present study. The model was able to reproduce 73% of the average nighttime Cl_2_ concentrations (Figure 1d).

#### 2.2.3 Chlorine-Initiated Secondary Organic Aerosols

In the default WRF-Chem model, the secondary organic aerosols (SOA) formation is initiated by the oxidation of VOCs, including OH^·^, O_3_, and NO_3_^·^, as well as the glyoxal uptake into aqueous aerosols to form SOA.^38,48^ The volatility basis set (VBS) framework is used to partition organic compounds between gas and particle phases based on their volatility. In this study, we added reactions involving chlorine-initiated SOA formation (R49–R72 in Table S1) into our model based on the work of Li et al.^4^ A detailed description of the SOA formation in the standard and revised WRF-Chem models can be found in that study.

### 2.3 Observations

The observational data were obtained from a coastal background site (22.21°N, 114.25°E, Cape D’Aguilar, Hong Kong, Figure 1b) between August 31 and October 7, 2018. The field study was conducted during the autumn season, when Hong Kong experiences prolonged photochemical pollution.^49^ Details of the campaign and observational data have been provided by Peng et al.^22^ The data, including Cl_2_, ClNO_2_, O_3_, NO_2_, PM_2.5_, and the components of the fine-mode aerosols, including nitrate, chloride, ammonia, and sulfate, were used to evaluate the model performance. Additionally, the observed meteorological data regarding wind speed, wind direction, and surface temperature along with data on air pollutants (O_3_, PM_2.5_, and NO_2_) at 54 official monitoring sites in South China, obtained from the China Ministry of Ecology and Environment (MEE) and HKEPD, were used for model validation.

### 2.4 Model Simulations

The simulation period is consistent with the field campaign at the Cape D’Aguilar site, with 7 days as spin-up (from August 24 to October 7, 2018). We conducted three major simulations, BASE, wCl_2_, and CL, the results of which are discussed in detail. The default setting in the BASE case includes no chlorine sources or chemistry; it includes only the reactions in the standard WRF-Chem configuration. The wCl_2_ case includes the chlorine sources and Cl_2_-related reactions described in the previous section. The difference between wCl_2_ and BASE represents the impact of photolytic Cl_2_ production. The CL case includes all chlorine sources and reactions (Dai et al.;^3^ Zhang et al.;^41^ this work; Table S1). The results of the CL case were used to validate the model’s performance. The difference between the CL and BASE cases represents the impact of all chlorine sources and reactions. We also conducted two additional sensitivity tests, namely BASE_50%EMIS and CL_50%EMIS, to evaluate the impact of reactive chlorine chemistry on O_3_ formation when anthropogenic emissions of acid gases (NO_*x*_ and SO_2_) are reduced by a factor of 2.

## 3 Results and Discussion

### 3.1 Abundance of Reactive Chlorine Species

Figure 1c shows the time series of the simulated and observed Cl_2_, ClNO_2_, O_3_, and fine particulate components, including chloride and nitrate, at the Cape D’Aguilar site during the field campaign period. The site received air masses from the South China Sea in the early part of the study (August 31 to September 4, 2018). A super typhoon named “Mangkhut” impacted the region from September 14 to September 20, 2018 (Figure S4a), during which the halogen instrument was switched off to prevent rainwater from entering it. The continental air masses prevailed at the site on other days (from September 4 to September 14 and from September 22 to October 7). During the continental outflow, moderate to high levels of O_3_ pollution were observed, with hourly O_3_ levels reaching 186 ppb on September 11; this indicates active photochemistry during the campaign period. The measured high levels of O_3_ frequently coincided with the daytime peak value of Cl_2_ (>400 pptv) and reached 998 pptv during the O_3_ episode, revealing a link between daytime Cl_2_ production and anthropogenic pollution.

The model-simulated Cl_2_ (in CL case) reasonably captured the hourly variations and daytime peak of the measured Cl_2_ at the Cape D’Aguilar site (Figure 1c, correlation coefficient = 0.72). The simulated concentration of Cl_2_ accounted for 71% and 73% of the average observed Cl_2_ during daytime [06:00 to 19:00 Local Standard Time (LST)] and nighttime (19:00 to 06:00 LST), respectively (Figure 1d). The simulation of ClNO_2_ also agreed with the magnitudes and temporal variations of the observed ClNO_2_, with an average underestimation of 24% (Figure 1e). The model also well simulated N_2_O_5,_ with a model-observation discrepancy within 15% (Figure S5), and specific VOC species.^40^ The simulated O_3_ and fine aerosols of particulate Cl^–^ and NO_3_^–^ at the site matched the hourly variations in the measurements reasonably well (Figure 1c), with average biases of 5% (or 3 ppbv), 12% (or 0.5 μg m^–3^), and −3% (or 0.5 μg m^–3^), respectively. The value of aerosol H^+^ was slightly underestimated at the site, with the simulated ranges of aerosol pH being 1.9–3.7 (against the observed range of 1.5–3.0; Figure S6a). The statistical parameters describing the performance of our model against the observations at the monitoring sites in South China are listed in Table S4 (SI Text S2). In summary, the modified WRF-Chem model with the updated chlorine mechanisms can reasonably reproduce the temporal variations in reactive chlorine species and air pollutants at the monitored coastal site in South China. As the continental air mass predominates in the autumn season and is associated with high concentrations of reactive halogens and secondary pollutants, we focus on the results related to continental air mass.

**Figure 2:**
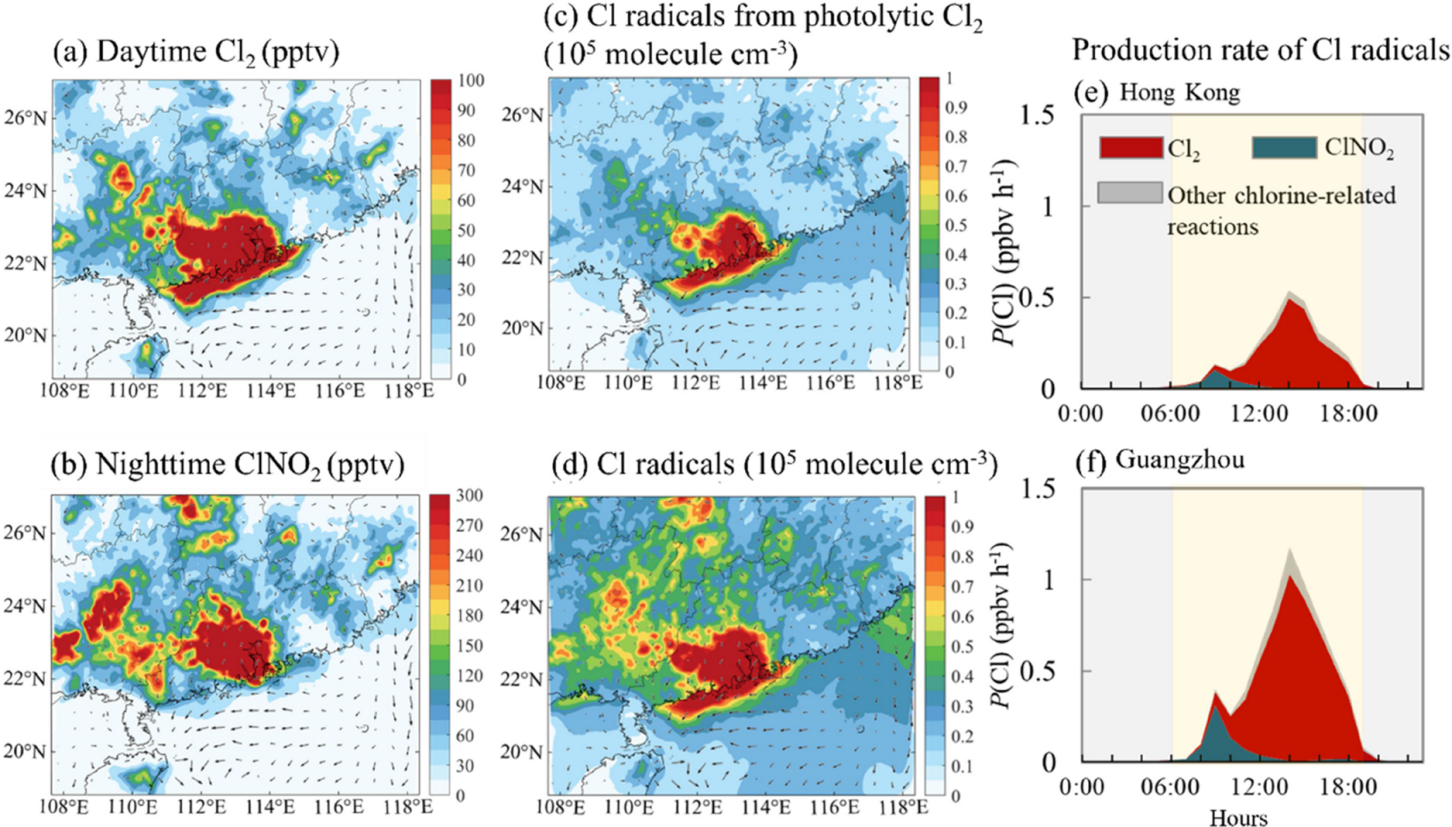
Average spatial distributions of the simulated mixing ratios of chlorine species (Cl_2_ and ClNO_2_) at the surface and their contributions to the production of Cl^·^ in continental air in two urban areas. (a and b) Average simulated spatial distribution of the mixing ratios (unit: pptv) of (a) Cl_2_ during daytime (06:00 to 19:00 LST) and (b) ClNO_2_ during nighttime (19:00 to 06:00 LST). (c and d) Impacts of (c) Cl_2_ photolysis and (d) all chlorine-related reactions to the mixing ratio of Cl^·^ (unit: 10^5^ molecules cm^–3^) during daytime in South China. (e, f) Contributions of photolytic Cl_2_, ClNO_2_, and reactions related to other chlorine species to the production rate of Cl^·^ [*P*(Cl); unit: ppbv h^–1^] in (e) Hong Kong and (f) Guangzhou. The arrows in panels (a–d) represent the scaled wind speed and wind direction. The period shaded in yellow and gray in panels (e and f) represents daytime and nighttime, respectively.

Figure 2a depicts the horizontal distributions of simulated Cl_2_ during the daytime (06:00 to 19:00 LST) in the surface air of South China for the continental air mass. Elevated concentrations of daytime Cl_2_ are predicted in the western part of Guangdong province (by up to 220 pptv), with a high concentration band along the coastal areas of South China. The presence of daytime Cl_2_ also extends 200–300 km into inland areas in Guangdong and Guangxi provinces (up to 80 pptv). The distribution of daytime Cl_2_ is affected by the distribution of its precursors, aerosol acidity, and meteorological conditions. During the field study period, the northeasterly offshore winds from inland to the South China Sea prevailed in South China, due to the high pressure over northern China (Figure S4b). Under this wind flow, a high concentration of fine NO_3_^–^ is predicted in the western and southern parts of South China and the adjacent marine areas (∼5 μg m^–3^; Figure S7a). The simulated aerosol pH value ranges from 1.5 to 3.2 in the coastal areas with high NO_3_^–^-loading (Figure S7b), providing sufficient acidity to facilitate the daytime Cl_2_ production. The high variation in daytime Cl_2_ across the coastal line may be attributable to the mesoscale land – sea breezes as well as the specific variation in the thermal-dynamic structure along the coast depicted in Figure S4c,d.

The spatial distribution of nocturnal (19:00 to 06:00 LST) ClNO_2_ is somewhat different from that of Cl_2_. As shown in Figure 2b, elevated ClNO_2_ mixing ratios are predicted in the areas with abundant Cl_2_ production, and the simulated ranges of ClNO_2_ are 160–480 pptv on average at night; by contrast, the presence of nocturnal ClNO_2_ extends 500–600 km into central China (Hunan and Jiangxi provinces; up to 200 pptv). The high concentrations of ClNO_2_ are consistent with the calculated high concentrations of N_2_O_5_ (>200 pptv; Figure S7c) and fine Cl^–^ (∼0.5 μg m^–3^; Figure S7d). With regard to other chlorine species (ClNO_3_, ClO, HOCl, and HCl), elevated values are predicted along the coast and adjacent oceanic areas (Figure S8), revealing a large spatial presence and strong impact of reactive chlorine species.

Figure 2c, d shows the spatial distribution of the simulated mixing ratio of daytime Cl^·^ in the surface air of South China. The distribution of Cl· produced from photolytic Cl_2_ largely resembles the distribution of daytime Cl_2_ (Figure 2c), with high values (reaching 1.0 × 10^5^ molecules cm^–3^) in the estuary of PRD region and along the coastline of South China. When considering the Cl^·^ generated from other chlorine-related reactions, more elevated levels of Cl^·^ are predicted in inland (up to 0.8 × 10^5^ molecules cm^–3^; Figure 2d) areas and over the open ocean (by up to 0.2 × 10^5^ molecules cm^–3^).

Figure 2e, f shows the diurnal variation in the production rates of Cl^·^ (*P*(Cl)) via the photolysis of Cl_2_ and ClNO_2_, as well as other chlorine-related reactions in two urban areas, Hong Kong and Guangzhou, in South China. In Hong Kong (Figure 2e), the calculated value of *P*(Cl) peaked at noon (at 14:00 LST) with a value of 0.62 ± 0.31 ppbv h^–1^, while a smaller peak is predicted in the early morning (at 07:00 LST; 0.36 ± 0.12 ppbv h^–1^). These two peaks of Cl^·^ are attributed to contributions from Cl_2_ and ClNO_2_, respectively. In Guangzhou, the values of these two peaks are 1.1 ± 0.67 and 0.54 ± 0.32 ppbv h^–1^ (Figure 2f), respectively. Throughout the day (06:00 to 19:00 LST), Cl_2_ photolysis is the primary source of Cl^·^, contributing 76% (Hong Kong) and 79% (Guangzhou) to the total *P*(Cl). ClNO_2_ photolysis contributes 20% (Hong Kong) and 18% (Guangzhou) to the integrated *P*(Cl) during the daytime (06:00 to 19:00 LST), with a dominant contribution (70 and 75% in Hong Kong and Guangzhou, respectively) in the early morning (06:00 to 09:00 LST). The contribution of other chlorine-related reactions to *P*(Cl) is minor (<4%). Our calculated value of *P*(Cl) is comparable to the value reported by Liu et al.^25^ (∼1.0 ppbv h^–1^) at a rural site in North China and is 1 order of magnitude higher than the values predicted by other global and regional models.^32,33^

### 3.2 Impact of Chlorine Chemistry on Atmospheric Oxidation

**Figure 3:**
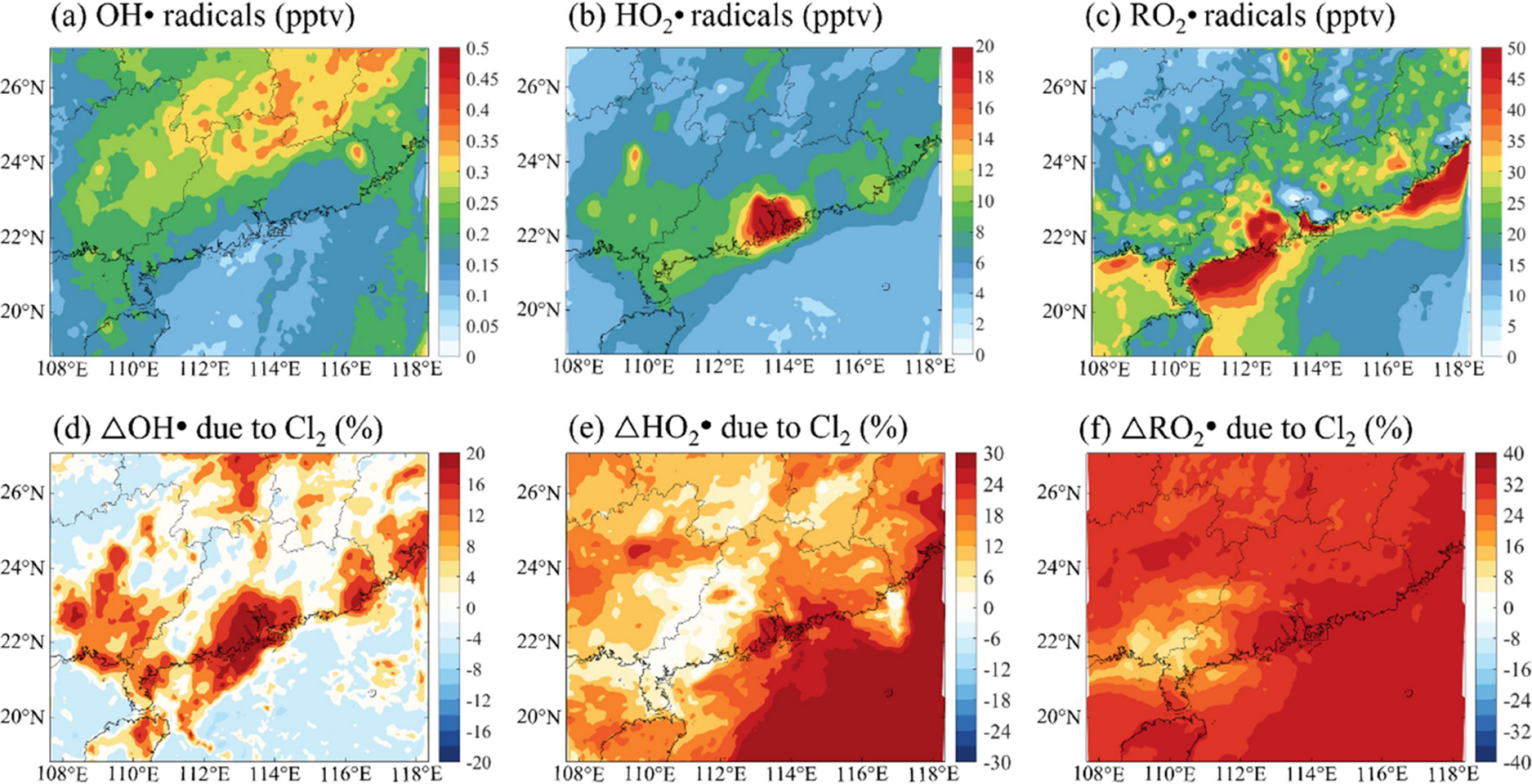
Impact of Cl_2_ on atmospheric oxidants in South China in continental air. (a–c) Average spatial distribution of simulated mixing ratios of (a) OH^·^, (b) HO_2_^·^, and (c) RO_2_^·^ (unit: pptv) with all chlorine sources and reactions (CL case) at the surface in continental air. (d–f) Percentage changes in simulated mixing ratios of OH^·^, HO_2_^·^, and RO_2_^·^ due to Cl^·^ produced from photolytic Cl_2_ during the daytime (Δchanges = (wCl_2_ case – BASE case)/BASE case × 100).

Cl^·^ can change the mixing ratios of conventional photochemical radicals, including OH^·^, HO_2_^·^, and RO_2_^·^. As shown in Figure 3d, with high levels of Cl^·^ from Cl_2_ photolysis, the surface mixing ratio of OH^·^ increases in the coastal and adjacent marine areas of the PRD region (by up to 30% or 0.1 pptv; Figure S9d). Increased levels of OH^·^ are also found in nearby coastal areas in Guangxi and Fujian provinces (∼0.06 pptv), where the anthropogenic NO_*x*_ emissions are high (Figure 1a). The mixing ratios of HO_2_^·^ and RO_2_^·^ in surface air are also increased by Cl_2_. As shown in Figure 3f, the levels of RO_2_^·^ increased throughout the domain due to the enhanced oxidation of VOCs by the Cl^·^ generated from photolytic Cl_2_, which further enhances the production of HO_2_^·^ (Figure 3e) and OH^·^ (Figure 3d) in the presence of NO_*x*_.^1,4,22^

The increases in the levels of RO_*x*_ (RO_*x*_ = OH^·^ + HO_2_^·^ + RO_2_^·^) due to Cl_2_ production partially explains the discrepancy between the observed and the underpredicted RO_*x*_ levels reported in model studies.^50^ Based on our results, the Cl_2_-induced increments in the levels of OH^·^, HO_2_^·^, and RO_2_^·^ are in the range of 3–20% (or 0.03–0.08 pptv; Figure S9a), 6–30% (or 1–5 pptv; Figure S9b) and 12–40% (or 2–10 pptv; Figure S9c), respectively. Regarding urban areas, the increments in RO_*x*_ radical levels range from 15 to 33% in Hong Kong (Figure S10a) and from 16 to 37% in Guangzhou (Figure S10b). These values are comparable to the 17 and 27% increases in HO_2_^·^ and RO_2_^·^ radical levels, respectively, at a polluted coastal site in South China^22^ and the 13 and 18% increments at a rural site in North China^25^ as well as the average increase of 12.5–33.5% in RO_*x*_ radical levels at an urban site in Beijing.^3^

A slight decrease in the OH· radical levels is predicted in the low-NO_*x*_ inland areas and the open ocean (4–6% or 0.015–0.02 pptv in Figure S9a) due to the destruction of O_3_ (the main source of OH^·^) by Cl^·^.^1^ Chlorine-induced heterogeneous changes in OH^·^ radical levels have also been reported in other modeling studies.^4,35,36^ The most distinct increases in the levels of HO_2_^·^ and RO_2_^·^ are predicted over the open ocean (>30% for HO_2_^·^ and >40% for RO_2_^·^), indicating a broad impact of photolytic Cl_2_ on atmospheric oxidants.

**Figure 4:**
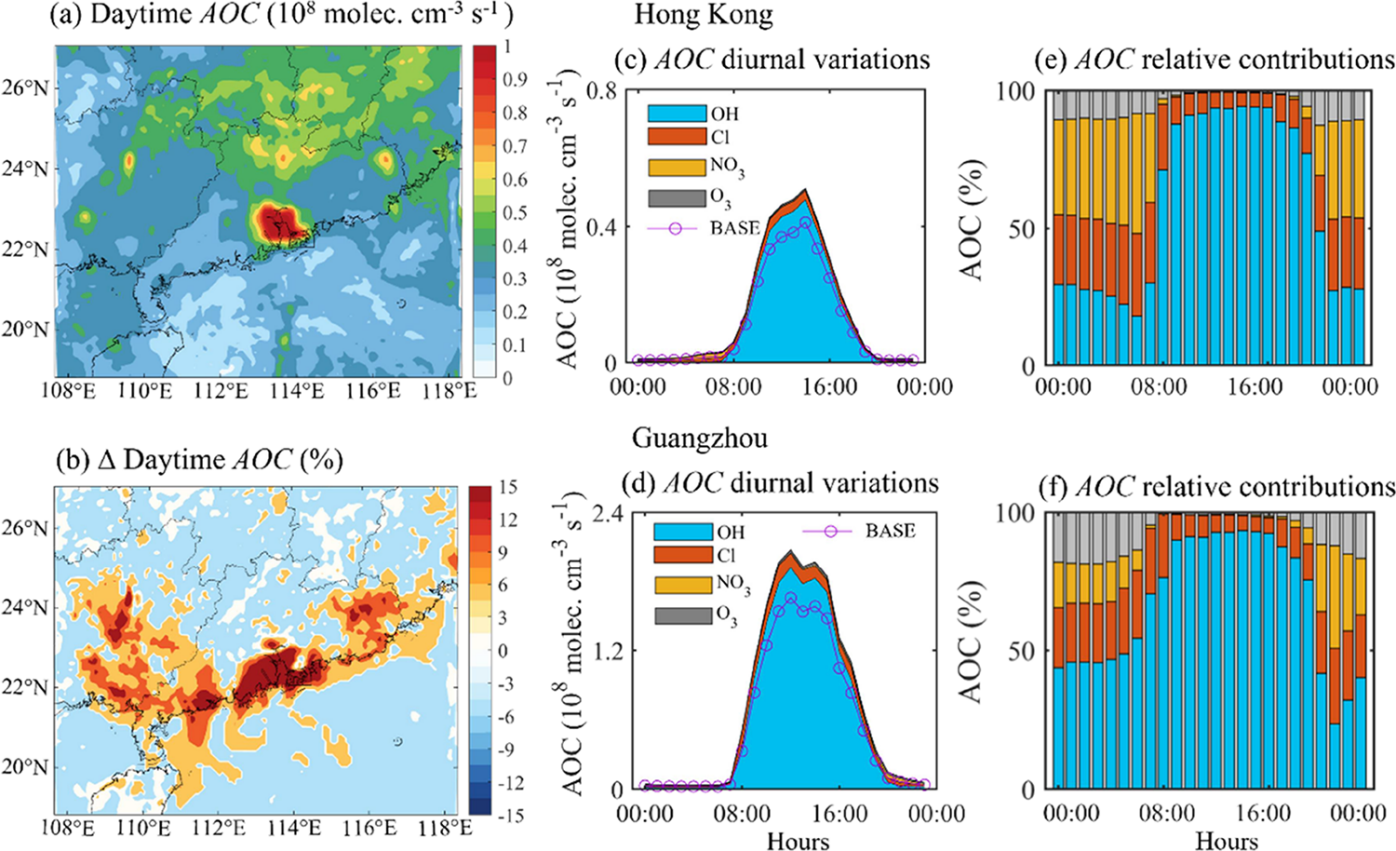
Impact of Cl^·^ on the daytime (06:00 to 19:00 LST) atmospheric oxidative capacity (AOC) in South China in continental air. (a) Simulated values of daytime AOC (unit: 10^8^ molecules cm^–3^ s^–1^; in CL case). (b) Percentage changes in the simulated values of daytime AOC due to Cl_2_ production. (c–f) Diurnal variations in and relative contributions of AOC at the monitoring sites in (c, e) Hong Kong and (d, f) Guangzhou.

The atmospheric oxidizing capacity (AOC) is a parameter that characterizes the self-cleansing ability of the atmosphere.^40^ It is used to derive the rate at which CO, CH_4_, and nonmethane hydrocarbons (NMHCs) are oxidized by atmospheric oxidants (see calculations in SI Text S3). In addition to the conventional oxidants, namely OH^·^, O_3_, and NO_3_^·^, Cl^·^ is also included in this study to evaluate its impact on AOC. As shown in Figure 4a, in the CL case, elevated values of average daytime (06:00 to 19:00 LST) AOC are predicted in the urban areas of the PRD regions (by up to 1.0 × 10^8^ molecules cm^–3^). The figure also shows relevant levels in inland (0.6 × 10^8^ molecules cm^–3^) and oceanic areas (0.2 × 10^8^ molecules cm^–3^). Cl_2_ increases daytime AOC in the high-NO_*x*_ coastal areas while reducing the AOC in relatively clean (both inland and the oceanic) areas (Figure 4b). The maximum increase in daytime AOC is predicted in the urban areas of the PRD (by 18–25% or 0.12–0.24 × 10^8^ molecules cm^–3^ s^–1^). The chlorine-induced change in daytime AOC is consistent with the change in the OH^·^ radical levels and the high levels of land-based CO (Figure S6e) and VOCs (Figure S11), which are the main reactors for calculating daytime AOC. The significant increase in daytime AOC is attributed to not only the chlorine-related reactions but also contributions from OH^·^-related and O_3_-related reactions, due to the impact of Cl^·^ on conventional radicals.

Figure 4c–f displays the diurnal variations and relative contributions of the AOC in Hong Kong and Guangzhou. When considering all chlorine-related reactions, the predicted peak daytime AOC increased by 25% (from 0.4 × 10^8^ to 0.5 × 10^8^ cm^–3^ s^–1^) in Hong Kong (Figure 4c) and by 30% (from 1.5 × 10^8^ to 2.0 × 10^8^ cm^–3^ s^–1^) in Guangzhou (Figure 4d). The chlorine-related reactions account for 10–15% and 8–12% of the daytime AOC in Hong Kong (Figure 4e) and Guangzhou (Figure 4f), respectively, with dominant contributions (>80%) from OH^·^-related reactions.

**Figure 5:**
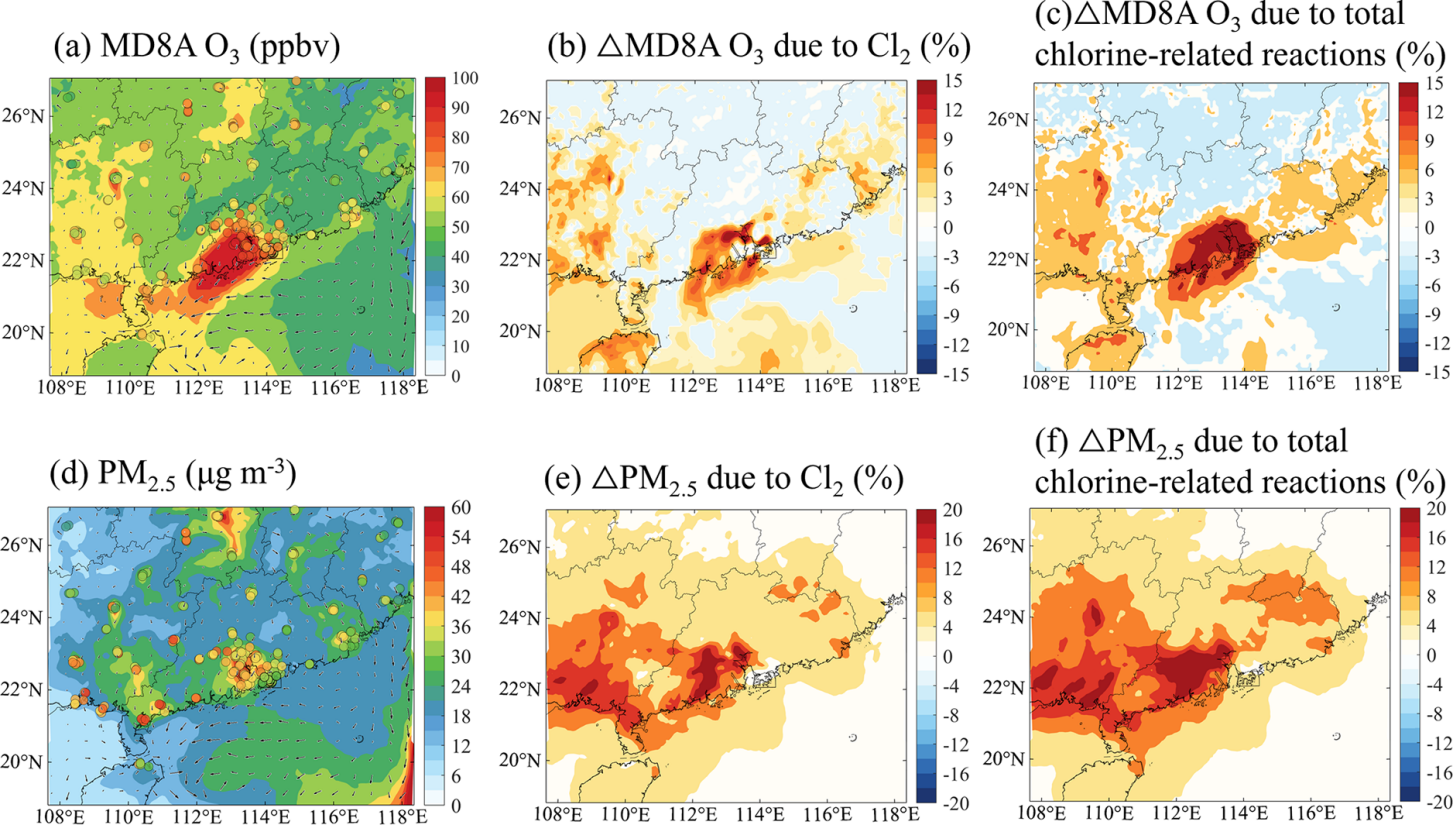
Impact of reactive chlorine species on the surface concentration of maximum daily 8-hour average (MD8A) O_3_ and fine aerosol in continental air. (a) Average simulated surface concentration of MD8A O_3_ (unit: ppbv) and (d) fine particulate matter (PM_2.5_; unit: μg m^–3^) in the CL case overplotted with the observed value for 54 monitoring sites in South China. (b–f) Percentage change in (b, c) MD8A O_3_ and (e, f) PM_2.5_ due to the impact of (b, e) Cl_2_ production (wCl_2_ case–BASE case) and (c, f) all chlorine-related reactions (CL case–BASE case); the arrows in panels (a, d) represent the scaled wind speed and wind direction.

Figure 5a–c shows the spatial distribution of maximum daily 8-hour average (MD8A) of O_3_ concentration at the surface and its changes due to the impact of chlorine chemistry. During the continental outflow, the O_3_ pollution (MD8A O_3_ mixing ratio >100 ppbv) is predicted in the western parts of the PRD region and adjacent marine areas (Figure 5a). The photolysis of Cl_2_ increases the simulated surface MD8A O_3_ concentration by 8% on average (Figure 5b) and by up to 12% (or 9 ppbv; Figure S12a) in the high-NO_*x*_ areas in South China. The areas with increased O_3_ levels increase coincided with those exhibiting increased peroxy radical levels and higher daytime AOC; this highlights the impact of high oxidative capacity on accelerating photochemical radical reactions and promoting O_3_ formation.^40^ Conversely, reductions in O_3_ concentration (by 3–4% or 1.5–2.0 ppbv) are predicted in relatively low-NO_*x*_ areas. The reactions of Cl^·^ with O_3_ and of ClO with NO_2_ can initiate the removal of O_3_ and NO_*x*_, thereby reducing O_3_ levels in relatively clean environments.^1,4,32^ Considering the impact of other chlorine-related reactions, the maximum O_3_ enhancement increased to 15% (or 11 ppbv, Figure 5c and S12b) in the polluted coastal areas, mainly due to the additional Cl^·^ produced via the photolysis of ClNO_2_.

The fine-particle concentrations are also enhanced through reactive chlorine chemistry. High levels of fine aerosols are predicted in the western parts of South China under continental outflow conditions (Figure 5d). With photolytic Cl_2_ production, the simulated concentrations of secondary aerosol increase throughout the domain by 10% on average, with the highest increase reaching 16% (or 8 μg m^–3^) in the western part of South China (Figure 5e). With regard to the secondary components (Figure S13), the maximum increase due to reactive chlorine species is 36% (or 2.2 μg m^–3^) for SOA, 21% (or 1.5 μg m^–3^) for nitrate, 10% (or 0.5 μg m^–3^) for sulfate, and 20% (or 1.5 μg m^–3^) for ammonium. The chlorine-induced increase in nitrate level is mainly due to the enhanced production rates from the reaction of NO_2_ with OH^·^ and through N_2_O_5_ hydrolysis. The modest increase in sulfate level results from the gaseous oxidation of SO_2_ by the increased amounts of OH· radicals and O_3_. The increase in ammonium level is due to the formation of ammonium nitrate (NH_4_NO_3_) through the reaction of NH_3_ with nitric acid (HNO_3_). SOA formation exhibits the largest increase, ascribed to increased levels of oxidants—including OH· and O_3_—and additional chlorine-initiated pathways that yield SOA. With other chlorine-related reactions, the increase in fine-aerosol concentration is 18% (10.2 μg m^–3^; Figure 5f).

The Cl_2_-induced increases in O_3_ and PM_2.5_ concentrations also partially explains the underpredicted secondary pollutant levels relative to the observed values in urban areas. As shown in Figure S14a, at the urban sites in Guangzhou, the average underestimation of the O_3_ level is lowered to 9% due to chlorine chemistry (from 30% in the BASE case), with dominant contributions by Cl_2_ production. For the simulation of PM_2.5_, the underprediction is also reduced by considering chlorine chemistry, from 12 to 20% (Figure S14b). These results demonstrate the importance of the previously overlooked reactive chlorine chemistry, especially that of Cl_2_, in simulations of O_3_ and aerosols in South China, indicating that reactive chlorine species can exacerbate secondary pollution in polluted coastal environments.

### 3.3 Impact of Chlorine Chemistry on O_3_-Formation Regimes

**Figure 6:**
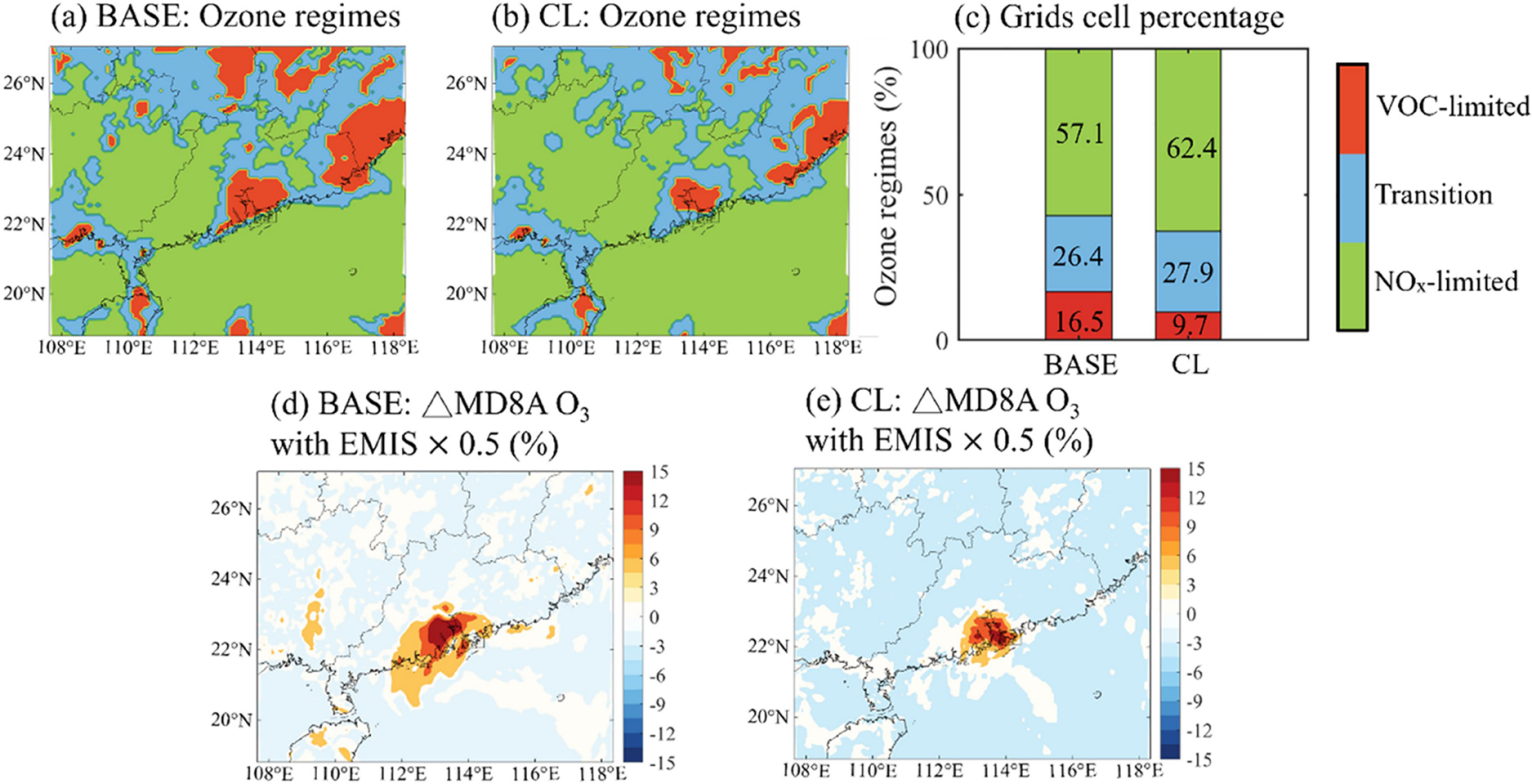
Impacts of reactive chlorine chemistry on O_3_ sensitive regimes and on O_3_ response to emission reduction. (a and b) O_3_ sensitive regimes in South China (a) in default case (BASE case) and (b) the case with all chlorine-related reactions (CL case). (c) Grid-cell proportions across the entire domain in the BASE and the CL cases. (d and e) Percentage changes in simulated concentrations of surface MD8A O_3_ due to (d) 50% reduction in NO_*x*_ and SO_2_ emission (BASE_50%EMIS case–BASE case) and (e) additional impact of reactive chlorine chemistry (CL_50%EMIS case–CL case).

The ratio of the production rates of H_2_O_2_ to that of HNO_3_ [*P*(H_2_O_2_)/*P*(HNO_3_)] is widely used as an indicator to quantify the sensitivity of O_3_ production to NO_*x*_ or VOCs.^36,51^ We adopt this indicator to determine the impact of reactive chlorine chemistry on the sensitivity of O_3_ to its precursors. As shown in Figure 6a, in the default condition (BASE case), the areas controlled by VOCs (VOC-limited, defined as *P*(H_2_O_2_)/*P*(HNO_3_) < 0.06) are located in the urban areas within the PRD and the coastal and inland areas with high NO_*x*_ emission. When considering all chlorine-related reactions (CL case; Figure 6b), the VOC-limited areas are suppressed and transformed into either transition (or mixed, i.e. controlling by both VOCs and NO_*x*_; 0.06 < *P*(H_2_O_2_)/*P*(HNO_3_) < 0.2) or NO_*x*_-limited (controlled by NO_*x*_; *P*(H_2_O_2_)/*P*(HNO_3_) > 0.2) areas. The changes in the O_3_ sensitivity regimes are related to the increase in the production of H_2_O_2_ due to the elevated HO_2_^·^ radical levels resulting from Cl_2_ production. Specifically, upon considering chlorine chemistry, the percentage of VOC-limited grid cells drops to 9.7% throughout the domain (from 16.5% in the BASE case, Figure 6c), whereas the percentages of NO_*x*_-limited and mixed-regime grid cells increase to 62.4% (from 57.1%) and 27.9% (from 26.4%), respectively. These changes indicate that reactive chlorine species tend to shift the O_3_ sensitivity from VOC-limited to mixed or NO_*x*_-limited regimes.

In China, a series of clean-air actions has been undertaken to mitigate anthropogenic emissions, leading to significant reductions in NO_*x*_ (28%) and SO_2_ (70%) emissions from 2013 to 2020.^52^ Further reductions are expected under future air-quality and low-carbon policies. An added benefit of reductions in NO_*x*_ and SO_2_ would be the simultaneous reduction of Cl_2_ production due to a decrease in nitrate, sulfate, and aerosol acidity. Simulations involving a 2-fold reduction in NO_*x*_ and SO_2_ emissions (CL_50%EMIS case) revealed significant reductions in surface Cl_2_ and ClNO_2_ levels (Figure S15), with the peak concentrations reduced to 120 and 220 pptv (from 220 and 480 pptv in CL case), respectively.

To assess the impact of chlorine chemistry on the response of O_3_ to emission reduction, another sensitivity analysis is conducted with a 50% reduction in NO_*x*_ and SO_2_ emissions in default condition (BASE_50%EMIS). As shown in Figure 6d, disregarding Cl chemistry caused the O_3_ concentration to increase in the coastal PRD and nearby marine areas (by up to 15% or 12 ppbv) due to the restricted NO_*x*_ titration of O_3_. When including chlorine chemistry, the increase in O_3_ level is restricted to a smaller fraction of the coastal PRD area, with a concurrent decline (to 10% or 8 ppbv; Figure 6e) in the extent of the increase. This weakened O_3_ increase is consistent with the shrinking of VOC-limited areas upon considering reactive chlorine chemistry. In NO_*x*_-limited areas, the O_3_ reduction increased to 5–6% (or 4.0–4.8 ppbv) as the areas become more sensitive to the reduction in NO_*x*_ emissions due to reactive chlorine chemistry.

Our results indicate that reactive chlorine chemistry tends to restrict the O_3_ increase in VOC-limited areas and augment the O_3_ reduction in NO_*x*_-limited areas. Thus, chlorine chemistry mitigates the negative impact of NO_*x*_ emissions reduction on O_3_ pollution while strengthening its benefit. Overlooking the impact of reactive chlorine chemistry on O_3_ formation would lead to an underestimation of the efficiency of NO_*x*_ emission reduction in mitigating O_3_ pollution in both urban and rural areas.

## 4 Summary and Implications

In this study, we implemented a parametrization of the production of daytime Cl_2_ from the photodissociation of particulate nitrate in acidic conditions and incorporated it into a regional air quality model. The updated model is able to explain a large fraction (∼70%) of the measured Cl_2_ at a polluted coastal site in South China. The photolysis of Cl_2_ is the dominant Cl· source, and it significantly increases the production of conventional radicals and secondary pollutants. Cl_2_-related chemistry also affects the designation of the O_3_-formation regimes and, thereby, the development of O_3_ control strategies. The parametrization implemented in our study may be applicable to other sites where elevated daytime nitrate concentrations and high aerosol acidity coexist.

Despite major improvements in the simulation of daytime Cl_2_ and its impact in our study, an average of 30% of the observed daytime Cl_2_ remains unexplained. The underestimation may be ascribable to additional chlorine sources or processes that were not considered in our study; these include the disinfectants used in water at wastewater treatment plants, hospitals, offices, and households,^53−55^ the contribution of iron-induced Cl_2_ production^36^ and other Cl_2_-production pathways,^26−28^ under-estimation of aerosol acidity, surface areas and mass (compared with the observations at the coastal site considered in this study), and other uncertainties in emissions, chemistry, and meteorology in the WRF-Chem model.^56−60^ Nonetheless, our study highlights the importance of daytime Cl_2_ in driving the atmospheric oxidation chemistry and the production of secondary pollutants on the South China coast and underscores the necessity of considering this daytime Cl_2_ in air quality models. Due to lack of observation of Cl_2_ in other areas the PRD, we could only validate our model against the Cl_2_ measurement at Cape D’Aguilar. Additional field measurements of Cl_2_ will be needed to evaluate the applicability of the Cl_2_ parametrization to other areas of the PRD (and beyond). We call for more field and modeling studies in other regions of China to obtain a more comprehensive picture of the geographical presence of Cl_2_ and its atmospheric impact.

## Author contribution

T.W. and J.D. designed the research. J.D. performed numerical experiments, analyzed the results, and created the figures. M.X. and W.S. performed the field observations. H.Q.S. ran the ISORROPIA-II and created the parametrizations for daytime Cl_2_. T.W. and J.D. wrote the manuscript. All authors reviewed and commented on the final paper.

## Supporting Information Available

Detailed information about chlorine-related reactions in the WRF-Chem model, calculation of hydrogen ions concentrations, and model evaluation (PDF)

## References

[ref1] Simpson W. R.; Brown S. S.; Saiz-Lopez A.; Thornton J. A.; von Glasow R. Tropospheric Halogen Chemistry: Sources, Cycling, and Impacts. Chem. Rev. 2015, 115 (10), 4035–4062. 10.1021/cr5006638. 25763598 PMC4469175

[ref2] Ma W.; Chen X.; Xia M.; Liu Y.; Wang Y.; Zhang Y.; Liu Y. Reactive Chlorine Species Advancing the Atmospheric Oxidation Capacities of Inland Urban Environments. Environ. Sci. Technol. 2023, 57 (39), 14638–14647. 10.1021/acs.est.3c05169. 37738177

[ref3] Dai J.; Liu Y.; Wang P.; Fu X.; Xia M.; Wang T. The impact of sea-salt chloride on O3 through heterogeneous reaction with N2O5 in a coastal region of south China. Atmos. Environ. 2020, 236, 117604 10.1016/j.atmosenv.2020.117604.

[ref4] Li Q.; Fu X.; Peng X.; Wang W.; Badia A.; Fernandez R. P.; Cuevas C. A.; Mu Y.; Chen J.; Jimenez J. L.; Wang T.; Saiz-Lopez A. Halogens Enhance Haze Pollution in China. Environ. Sci. Technol. 2021, 55 (20), 13625–13637. 10.1021/acs.est.1c01949. 34591460 PMC8529710

[ref5] Tanaka P. L.; Riemer D. D.; Chang S.; Yarwood G.; Mcdonald-Buller E. C.; Apel E. C. Direct evidence for chlorine-enhanced urban O3 formation in Houston, Texas. Atmos. Environ 2003, 37 (9–10), 1393–1400. 10.1016/S1352-2310(02)01007-5.

[ref6] Aschmann S. M.; Atkinson R. Rate constants for the gas-phase reactions of alkanes with Cl radicals at 296 ± 2 K. Int. J. Chem. Kinet. 1995, 27 (6), 613–622. 10.1002/kin.550270611.

[ref7] Nelson L.; Rattigan O. Absolute and relative rate constants for the reactions of hydroxyl radicals and chlorine radicals with a series of aliphatic alcohols and ethers at 298 K. Int. J. Chem. Kinet. 2010, 22, 1111–1126. 10.1002/kin.550221102.

[ref8] Li Q.; Fernandez R. P.; Hossaini R.; Iglesias-Suarez F.; Cuevas C. A.; Apel E. C.; Kinnison D. E.; Lamarque J. F.; Saiz-Lopez A. Reactive Halogens Increase the Global Methane Lifetime and Radiative Forcing in the 21st Century. Nat. Commun. 2022, 13, 2768. 10.1038/s41467-022-30456-8 35589794 PMC9120080

[ref9] Li Q.; Meidan D.; Hess P.; Añel J. A.; Cuevas C. A.; Doney S.; Fernandez R. P.; van Herpen M.; Höglund-Isaksson L.; Johnson M. S.; Kinnison D. E.; Lamarque J.-F.; Röckmann T.; Mahowald N. M.; Saiz-Lopez A. Global Environmental Implications of Atmospheric Methane Removal through Chlorine-mediated Chemistry-climate Interactions. Nat. Commun. 2023, 14, 4045 10.1038/s41467-023-39794-7. 37422475 PMC10329632

[ref10] Keene W. C.; Maben J. R.; Pszenny A. A.; Galloway J. N. Measurement technique for inorganic chlorine gases in the marine boundary layer. Environ. Sci. Technol. 1993, 27, 866–874. 10.1021/es00042a008.

[ref11] Thornton J. A.; Kercher J. P.; Riedel T. P.; Wagner N. L.; Cozic J.; Holloway J. S.; Dube W. P.; Wolfe G. M.; Quinn P. K.; Middlebrook A. M.; Alexander B.; Brown S. S. A large atomic chlorine source inferred from mid-continental reactive nitrogen chemistry. Nature 2010, 464, 271–274. 10.1038/nature08905. 20220847

[ref12] Mielke L. H.; Furgeson A.; Osthoff H. D. Observation of ClNO2 in a mid-continental urban environment. Environ. Sci. Technol. 2011, 45, 8889–8896. 10.1021/es201955u. 21877701

[ref13] Tham Y. J.; Wang Z.; Li Q.; Yun H.; Wang W.; Wang X.; Xue L.; Lu K.; Ma N.; Bohn B.; Li X.; Kecorius S.; Größ J.; Shao M.; Wiedensohler A.; Zhang Y.; Wang T. Significant concentrations of nitryl chloride sustained in the morning: Investigations of the causes and impacts on O3 production in a polluted region of northern China. Atmos. Chem. Phys. 2016, 16 (23), 14959–14977. 10.5194/acp-16-14959-2016.

[ref14] Osthoff H. D.; Roberts J. M.; Ravishankara A. R.; Williams E. J.; Lerner B. M.; Sommariva R.; Bates T. S.; Coffman D.; Quinn P. K.; Dibb J. E.; Stark H.; Burkholder J. B.; Talukdar R. K.; Meagher J.; Fehsenfeld F. C.; Brown S. S. High levels of nitryl chloride in the polluted subtropical marine boundary layer. Nat. Geosci. 2008, 1, 324–328. 10.1038/ngeo177.

[ref15] Xia M.; Peng X.; Wang W.; Yu C.; Sun P.; Li Y.; Liu Y.; Xu Z.; Wang Z.; Xu Z.; Nie W.; Ding A.; Wang T. Significant production of ClNO2 and possible source of Cl2 from N2O5 uptake at a suburban site in eastern China. Atmos. Chem. Phys. 2020, 20, 6147–6158. 10.5194/acp-20-6147-2020.

[ref16] Wang T.; Tham Y. J.; Xue L.; Li Q.; Zha Q.; Wang Z.; Poon S. C.; Dubé W. P.; Blake D. R.; Louie P. K. Observations of nitryl chloride and modeling its source and effect on O3 in the planetary boundary layer of South China. J. Geophys. Res.: Atmos. 2016, 121, 2476–2489. 10.1002/2015JD024556.

[ref17] Liao J.; Huey L. G.; Liu Z.; Tanner D. J.; Cantrell C. A.; Orlando J. J.; Flocke F. M.; Shepson P. B.; Weinheimer A. J.; Hall S. R.; Ullmann K.; Beine H. J.; Wang Y.; Ingall E. D.; Stephens C. R.; Hornbrook R. S.; Apel E. C.; Riemer D.; Fried A.; Mauldin R. L. III; Smith J. N.; Staebler R. M.; Neuman J. A.; Nowak J. B. High levels of molecular chlorine in the Arctic atmosphere. Nat. Geosci. 2014, 7, 91 10.1038/ngeo2046.

[ref18] McNamara S. M.; Raso A. R.; Wang S.; Thanekar S.; Boone E. J.; Kolesar K. R.; Peterson P. K.; Simpson W. R.; Fuentes J. D.; Shepson P. B. Springtime Nitrogen Oxide-Influenced Chlorine Chemistry in the Coastal Arctic. Environ. Sci. Technol. 2019, 53, 8057–8067. 10.1021/acs.est.9b01797. 31184868

[ref19] Spicer C. W.; Chapman E. G.; Finlayson-Pitts B. J.; Plastridge R. A.; Hubbe J. M.; Fast J. D.; Berkowitz C. M. Unexpectedly high concentrations of molecular chlorine in coastal air. Nature 1998, 394, 353–356. 10.1038/28584.

[ref20] Lawler M. J.; Sander R.; Carpenter L. J.; Lee J. D.; Von Glasow R.; Sommariva R.; Saltzman E. S. HOCl and Cl2 observations in marine air. Atmos. Chem. Phys. 2011, 11, 7617–7628. 10.5194/acp-11-7617-2011.

[ref21] Riedel T. P.; Bertram T. H.; Crisp T. A.; Williams E. J.; Lerner B. M.; Vlasenko A.; Li S. M.; Gilman J.; de Gouw J.; Bon D. M.; Wagner N. L.; Brown S. S.; Thornton J. A. Nitryl chloride and molecular chlorine in the coastal marine boundary layer. Environ. Sci. Technol. 2012, 46, 10463–10470. 10.1021/es204632r. 22443276

[ref22] Peng X.; Wang T.; Wang W.; Ravishankara A. R.; George C.; Xia M.; Cai M.; Li Q.; Salvador C. M.; Lau C.; Lyu X.; Poon C. N.; Mellouki A.; Mu Y.; Hallquist M.; Saiz-Lopez A.; Guo H.; Herrmann H.; Yu C.; Dai J.; Wang Y.; Wang X.; Yu A.; Leung K.; Lee S.; Chen J. Photodissociation of particulate nitrate as a source of daytime tropospheric Cl2. Nat. Commun. 2022, 13 (1), 939 10.1038/s41467-022-28383-9. 35177585 PMC8854671

[ref23] Li F.; Huang D. D.; Nie W.; Tham Y. J.; Lou S.; Li Y.; Tian L.; Liu Y.; Zhou M.; Wang H.; Qiao L.; Wang H.; Wang Z.; Huang C.; Li Y. J. Observation of nitrogen oxide-influenced chlorine chemistry and source analysis of Cl2 in the Yangtze River Delta. China. Atmos. Environ. 2023, 306, 119829 10.1016/j.atmosenv.2023.119829.

[ref24] Riedel T. P.; Wagner N. L.; Dubé W. P.; Middlebrook A. M.; Young C. J.; Öztürk F.; Bahreini R.; VandenBoer T. C.; Wolfe D. E.; Williams E. J. Chlorine activation within urban or power plant plumes: Vertically resolved ClNO2 and Cl2 measurements from a tall tower in a polluted continental setting. J. Geophys. Res.: Atmospheres 2013, 118, 8702–8715. 10.1002/jgrd.50637.

[ref25] Liu X.; Qu H.; Huey L. G.; Wang Y.; Sjostedt S.; Zeng L.; Lu K.; Wu Y.; Hu M.; Shao M.; Zhu T.; Zhang Y. High levels of daytime molecular chlorine and nitryl chloride at a rural site on the North China Plain. Environ. Sci. Technol. 2017, 51, 9588–9595. 10.1021/acs.est.7b03039. 28806070

[ref26] Roberts J. M.; Osthoff H. D.; Brown S. S.; Ravishankara A. N2O5 oxidizes chloride to Cl2 in acidic atmospheric aerosol. Science 2008, 321, 1059–1059. 10.1126/science.1158777. 18599742

[ref27] Roberts J. M.; Osthoff H. D.; Brown S. S.; Ravishankara A.; Coffman D.; Quinn P.; Bates T. Laboratory studies of products of N2O5 uptake on Cl–containing substrates. Geophys. Res. Lett. 2009, 36 (20), L20808 10.1029/2009GL040448.

[ref28] Deiber G.; George Ch.; Le Calvé S.; Schweitzer F.; Mirabel Ph. Uptake study of ClONO2 and BrONO2 by Halide containing droplets. Atmos. Chem. Phys. 2004, 4, 1291–1299. 10.5194/acp-4-1291-2004.

[ref29] Knipping E. M.; Lakin M. J.; Foster K. L.; Jungwirth P.; Tobias D. J.; Gerber R. B.; Dabdub D.; Finlayson-Pitts B. J. Experiments and simulations of ion-enhanced interfacial chemistry on aqueous NaCl aerosols. Science 2000, 288 (2000), 301–306. 10.1126/science.288.5464.301. 10764637

[ref30] Van-Herpen M. M. J. W.; Li Q.; Saiz-Lopez A.; Liisberg J. B.; Röckmann T.; Cuevas C. A.; Fernandez R. P.; Mak J. E.; Mahowald N. M.; Hess P.; Meidan D.; Stuut J.-B. W.; Johnson M. Photocatalytic Chlorine Atom Production on Mineral Dust–Sea Spray Aerosols over the North Atlantic. Proc. Natl. Acad. Sci. U.S.A. 2023, 120 (31), e2303974120 10.1073/pnas.2303974120. 37487065 PMC10400977

[ref31] Faxon C. B.; Bean J. K.; Ruiz L. H. Inland concentrations of Cl2 and ClNO2 in southeast Texas suggest chlorine chemistry significantly contributes to atmospheric reactivity. Atmosphere 2015, 6, 1487–1506. 10.3390/atmos6101487.

[ref32] Yi X.; Sarwar G.; Bian J. T.; Huang L.; Li Q. Y.; Jiang S.; Liu H. Q.; Wang Y. J.; Chen H.; Wang T.; Chen J. M.; Saiz-Lopez A.; Wong D. C.; Li L. Significant impact of reactive chlorine on complex air pollution over the Yangtze River Delta region, China. J. Geophys. Res: Atmospheres 2023, 128, e2023JD038898 10.1029/2023JD038898. PMC11864322 40013108

[ref33] Qiu X.; Ying Q.; Wang S.; Duan L.; Wang Y.; Lu K.; Wang P.; Xing J.; Zheng M.; Zhao M.; Zheng H.; Zhang Y.; Hao J. Significant impact of heterogeneous reactions of reactive chlorine species on summertime atmospheric O3 and free-radical formation in north China. Sci. Total Environ. 2019, 693, 133580 10.1016/j.scitotenv.2019.133580. 31376754

[ref34] Chen Q.; Xia M.; Peng X.; Yu C.; Sun P.; Li Y.; Liu Y.; Xu Z.; Xu Z.; Wu R.; Nie W.; Ding A.; Zhao Y.; Wang T. Large daytime molecular chlorine missing source at a suburban site in East China. J. Geophys. Res. Atmos. 2022, 127, e2021JD035796 10.1029/2021JD035796.

[ref35] Wang X.; Jacob D. J.; Eastham S. D.; Sulprizio M. P.; Zhu L.; Chen Q.; Alexander B.; Sherwin T.; Evans M. J.; Lee B. H.; Haskins J. D.; Lopez-Hilfiker F. D.; Thornton J. A.; Huey G. L.; Liao H. The role of chlorine in global tropospheric chemistry. Atmos. Chem. Phys. 2019, 19, 3981–4003. 10.5194/acp-19-3981-2019.

[ref36] Chen Q.; Wang X.; Fu X.; Li X.; Alexander B.; Peng X.; Wang T. Impact of Molecular Chlorine Production from Aerosol Iron Photochemistry on Atmospheric Oxidative Capacity in North China. Environ. Sci. Technol. 2024, 58, 12585–12597. 10.1021/acs.est.4c02534. 38956968

[ref37] Skamarock W. C.; Klemp J. B.; Dudhia J.; Gill D. O.; Liu Z.; Berner J.; Wang W.; Powers J. G.; Duda M. G.; Barker D. M.; Huang X.-Y. A Description of the Advanced Research WRF Model Version 4; Tech. rep.; UCAR/NCAR, 2019.

[ref38] Emmons L. K.; Walters S.; Hess P. G.; Lamarque J.-F.; Pfister G. G.; Fillmore D.; Granier C.; Guenther A.; Kinnison D.; Laepple T.; Orlando J.; Tie X.; Tyndall G.; Widmeyer C.; Baughcum S. L.; Kloster S. Description and evaluation of the Model for O3 and Related chemical Tracers, version 4 (MOZART-4). Geosci. Model Dev. 2010, 3, 43–67. 10.5194/gmd-3-43-2010.

[ref39] Zaveri R. A.; Easter R. C.; Fast J. D.; Peters L. K. Model for Simulating Aerosol Interactions and Chemistry (MOSAIC). J. Geophys. Res. 2008, 113 (13), D13204 10.1029/2007jd008782.

[ref40] Dai J.; Brasseur G. P.; Vrekoussis M.; Kanakidou M.; Qu K.; Zhang Y.; Zhang H.; Wang T. The atmospheric oxidizing capacity in China–Part 1: Roles of different photochemical processes. Atmos. Chem. Phys. 2023, 23, 14127–14158. 10.5194/acp-23-14127-2023.

[ref41] Zhang L.; Li Q.; Wang T.; Ahmadov R.; Zhang Q.; Li M.; Lv M. Combined impacts of nitrous acid and nitryl chloride on lower-tropospheric O3: new module development in WRF-Chem and application to China. Atmos. Chem. Phys. 2017, 17, 9733–9750. 10.5194/acp-17-9733-2017.

[ref42] Huang Z. J.; Zhong Z. M.; Sha Q. G.; Xu Y. Q.; Zhang Z. W.; Wu L. L.; Wang Y. Z.; Zhang L. H.; Cui X. Z.; Tang M. S.; Shi B. W.; Zheng C. Z.; Li Z.; Hu M. M.; Bi L. L.; Zheng J. Y.; Yan M. An updated model-ready emission inventory for Guangdong Province by incorporating big data and mapping onto multiple chemical mechanisms. Sci. Total Environ. 2021, 769, 144535 10.1016/j.scitotenv.2020.144535. 33486173

[ref43] Fu X.; Wang T.; Wang S.; Zhang L.; Cai S.; Xing J.; Hao J. Anthropogenic emissions of hydrogen chloride and fine particulate chloride in China. Environ. Sci. Technol. 2018, 52 (3), 1644–1654. 10.1021/acs.est.7b05030. 29376646

[ref44] Dalton E. Z.; Hoffmann E. H.; Schaefer T.; Tilgner A.; Herrmann H.; Raff J. D. Daytime Atmospheric Halogen Cycling through Aqueous-Phase Oxygen Atom Chemistry. J. Am. Chem. Soc. 2023, 145 (29), 15652–15657. 10.1021/jacs.3c03112. 37462273

[ref45] Xia M.; Wang T.; Wang Z.; Chen Y.; Peng X.; Huo Y.; Wang W.; Yuan Q.; Jiang Y.; Guo H.; Lau C.; Leung K.; Yu A.; Lee S. Pollution-Derived Br_2_ Boosts Oxidation Power of the Coastal Atmosphere. Environ. Sci. Technol. 2022, 56 (17), 12055–12065. 10.1021/acs.est.2c02434. 35948027

[ref46] Chen X.; Xia M.; Wang W.; Yun H.; Yue D.; Wang T. Fast near-surface ClNO_2_ production and its impact on O3 formation 656 during a heavy pollution event in South China. Sci. Total Environ. 2023, 858, 159998 10.1016/j.scitotenv.2022.159998. 36368396

[ref47] Yang X.; Wang T.; Xia M.; Gao X.; Li Q.; Zhang N.; Gao Y.; Lee S.; Wang X.; Xue L.; Yang L.; Wang W. Abundance and origin of fine particulate chloride in continental China. Sci. Total Environ. 2018, 624, 1041–1051. 10.1016/j.scitotenv.2017.12.205. 29929221

[ref48] Knote C.; Hodzic A.; Jimenez J. L.; Volkamer R.; Orlando J. J.; Baidar S.; Brioude J.; Fast J.; Gentner D. R.; Goldstein A. H.; Hayes P. L.; Knighton W. B.; Oetjen H.; Setyan A.; Stark H.; Thalman R.; Tyndall G.; Washenfelder R.; Waxman E.; Zhang Q. Simulation of semi-explicit mechanisms of SOA formation from glyoxal in aerosol in a 3-D model. Atmos. Chem. Phys. 2014, 14 (12), 6213–6239. 10.5194/acp-14-6213-2014.

[ref49] Wang T.; Dai J.; Lam K. S.; Nan Poon C.; Brasseur G. P. Twenty-five years of lower tropospheric O3 observations in tropical East Asia: The influence of emissions and weather patterns. Geophys. Res. Lett. 2019, 46, 11463–11470. 10.1029/2019GL084459.

[ref50] Tan Z.; Lu K.; Jiang M.; Su R.; Wang H.; Lou S.; Fu Q.; Zhai C.; Tan Q.; Yue D.; Chen D.; Wang Z.; Xie S.; Zeng L.; Zhang Y. Daytime atmospheric oxidation capacity in four Chinese megacities during the photochemically polluted season: a case study based on box model simulation. Atmos. Chem. Phys. 2019, 19 (6), 3493–3513. 10.5194/acp-19-3493-2019.

[ref51] Tonnesen G. S.; Dennis R. L. Analysis of radical propagation efficiency to assess ozone sensitivity to hydrocarbons and NO x: 2. Long-lived species as indicators of ozone concentration sensitivity. J. Geophys. Res: Atmos. 2000, 105 (D7), 9227–9241. 10.1029/1999jd900372.

[ref52] Wang T.; Xue L. K.; Feng Z. Z.; Dai J. N.; Zhang Y. N.; Tan Y. Ground level O3 pollution in China: a synthesis of recent findings on influencing factors and impacts. Environ. Res. Lett. 2022, 17 (6), 063003 10.1088/1748-9326/ac69fe.

[ref53] Yin S.; Yi X.; Li L.; Huang L.; Ooi M. C. G.; Wang Y.; Allen D. T.; Streets D. G. An updated anthropogenic emission inventory of reactive chlorine precursors in China. ACS Earth Space Chem. 2022, 6 (7), 1846–1857. 10.1021/acsearthspacechem.2c00096.

[ref54] Hong Y.; Liu Y.; Chen X.; Fan Q.; Chen C.; Chen X.; Wang M. The role of anthropogenic chlorine emission in surface ozone formation during different seasons over eastern China. Sci. Total Environ. 2020, 723, 137697 10.1016/j.scitotenv.2020.137697. 32392687

[ref55] Yi X.; Yin S.; Huang L.; Li H.; Wang Y.; Wang Q.; Chan A.; Traoré D.; Ooi M. C. G.; Chen Y.; Allen D. T.; Li L. Anthropogenic emissions of atomic chlorine precursors in the Yangtze River Delta region, China. Sci. Total Environ. 2021, 771, 144644 10.1016/j.scitotenv.2020.144644. 33736175

[ref56] Zhang L.; Chen Y.; Zhao Y.; Henze D. K.; Zhu L.; Song Y.; Paulot F.; Liu X.; Pan Y.; Lin Y.; Huang B. Agricultural Ammonia Emissions in China: Reconciling Bottom-up and Top-down Estimates. Atmos. Chem. Phys. 2018, 18 (1), 339–355. 10.5194/acp-18-339-2018.

[ref57] Nault B. A.; Jo D. S.; McDonald B. C.; Campuzano-Jost P.; Day D. A.; Hu W.; Schroder J. C.; Allan J.; Blake D. R.; Canagaratna M. R.; Coe H.; Coggon M. M.; DeCarlo P. F.; Diskin G. S.; Dunmore R.; Flocke F.; Fried A.; Gilman J. B.; Gkatzelis G.; Hamilton J. F.; Hanisco T. F.; Hayes P. L.; Henze D. K.; Hodzic A.; Hopkins J.; Hu M.; Huey L. G.; Jobson B. T.; Kuster W. C.; Lewis A.; Li M.; Liao J.; Nawaz M. O.; Pollack I. B.; Peischl J.; Rappenglück B.; Reeves C. E.; Richter D.; Roberts J. M.; Ryerson T. B.; Shao M.; Sommers J. M.; Walega J.; Warneke C.; Weibring P.; Wolfe G. M.; Young D. E.; Yuan B.; Zhang Q.; de Gouw J. A.; Jimenez J. L. Secondary organic aerosols from anthropogenic volatile organic compounds contribute substantially to air pollution mortality. Atmos. Chem. Phys. 2021, 21, 11201–11224. 10.5194/acp-21-11201-2021.

[ref58] Tao W.; Su H.; Zheng G.; Wang J.; Wei C.; Liu L.; Ma N.; Li M.; Zhang Q.; Pöschl U.; Cheng Y. Aerosol pH and Chemical Regimes of Sulfate Formation in Aerosol Water during Winter Haze in the North China Plain. Atmos. Chem. Phys. 2020, 20 (20), 11729–11746. 10.5194/acp-20-11729-2020.

[ref59] Ruan X.; Zhao C.; Zaveri R. A.; He P.; Wang X.; Shao J.; Geng L. Simulations of aerosol pH in China using WRF-Chem (v4.0): sensitivities of aerosol pH and its temporal variations during haze episodes. Geosci. Model Dev. 2022, 15, 6143–6164. 10.5194/gmd-15-6143-2022.

[ref60] Craig R. L.; Peterson P. K.; Nandy L.; Lei Z.; Hossain M. A.; Camarena S.; Dodson R. A.; Cook R. D.; Dutcher C. S.; Ault A. P. Direct Determination of Aerosol pH: Size-Resolved Measurements of Submicrometer and Supermicrometer Aqueous Particles. Anal. Chem. 2018, 90 (19), 11232–11239. 10.1021/acs.analchem.8b00586. 30203960

[ref61] Dai J.; Wang T. Impact of international shipping emissions on ozone and PM_2.5_ in East Asia during summer: the important role of HONO and ClNO_2_. Atmos. Chem. Phys. 2021, 21, 8747–8759. 10.5194/acp-21-8747-2021.

[ref62] Zhang L.; Wang T.; Zhang Q.; Zheng J.; Xu Z.; Lv M. Potential sources of nitrous acid (HONO) and their impacts on ozone: A WRF-Chem study in a polluted subtropical region. J. Geophys. Res. Atmos. 2016, 121 (7), 3645–3662. 10.1002/2015JD024468.

