# Supplementary material for: Significant Impact of a Daytime Halogen Oxidant on Coastal Air Quality
Source: Environ Sci Technol. 2025 Jan 24;59(4):2169–80. doi: 10.1021/acs.est.4c08360 (PMC11800395; doi:10.1021/acs.est.4c08360)
Supplement: Supplementary file 1 — es4c08360_si_001.pdf [file es4c08360_si_001.pdf]

Supplementary Materials for

Significant impact of a daytime halogen oxidant on coastal air quality

Jianing Dai[1], Tao Wang[1]*, Hengqing Shen[1], Men Xia[4,5], Weihang Sun[1], Guy P. Brasseur[1,2,3]

[1] Department of Civil and Environmental Engineering, The Hong Kong Polytechnic University, Hong Kong SAR 999077, China

[2] Environmental Modelling Group, Max Planck Institute for Meteorology, Hamburg, 20146, Germany

[3] NSF-National Center for Atmospheric Research, Boulder, Colorado, 80307, USA

[4] Institute for Atmospheric and Earth System Research/Physics, Faculty of Science, University of Helsinki, Helsinki 00014, Finland

[5] Aerosol and Haze Laboratory, Beijing Advanced Innovation Center for Soft Matter Science and Engineering, Beijing University of Chemical Technology, Beijing 100029, China

* Correspondence to: Tao Wang (tao.wang@polyu.edu.hk)

Contents:

Supplementary Text S1 to S3

S1. ISORROPIA model

S2. Model performance.

S3. The Atmospheric Oxidative Capacity.

Supplementary Figures S1 to S15

Supplementary Tables S1 to S4

Supplementary References

Supplementary Text

S1. ISORROPIA model. The aqueous-phase concentration of aerosol H^+ ($[H^+]$, unit: mol L^{-1}) was calculated using the ISORROPIA-II model.[1,2] The model inputs are hourly measurements of ambient relative humidity, molar concentrations (unit: mol m^{-3}) of fine aerosol of particulate Cl^-, NO_3^-, SO_4^{2-}, Na^+, and NH_4^+, measured by an ion chromatography (MARGA), and gas-phase ammonia. Aerosol pH was calculated as $-\log_{10}([H^+])$.

S2. Model Performance. Table S3 shows the statistics evaluation of our model performance for meteorological parameters (surface temperature, wind speed, and relative humidity) and air pollutants (NO_2, SO_2, CO, O_3, and $PM_{2.5}$) at regular monitoring sites in South China. Based on the statistics, our simulated meteorological parameters match well with the observations, with relatively low mean bias (< 2%) and high correlation coefficient (> 90%). For air pollutants, a slight underestimation in surface ozone concentration is simulated in South China (Figure 5a), with the mean bias of -5.5 ppbv (or 12%) on average, mainly located in the non-urban areas. This underestimated ozone concentration is relevant to the underestimated NO_2 and CO concentrations in low-NO_x areas (Figure S6c-d), as the precursors to ozone production. Other reasons for the model discrepancies can be the uncertainties lies in land-used data,[3] natural and anthropogenic emissions,[4,5] and NO_2-related parameterizations used in the model.[6] For the concentration of $PM_{2.5}$, a slight underestimation is calculated in southern and western part of South China (Figure 5d), with the mean bias of 6.5 μg m^{-3} (or 13%) on average in entire domain. A slight overestimation of $PM_{2.5}$ is distributed on the western coast of South China (Figure 5d), which may be attributable to the uncertainties of sea-salt aerosol deposition in models.[7]

In summary, our model performance of meteorological conditions and the air pollutants are in generally good agreement with the observations in South China. Our results are reliable to conduct further analysis on the impacts of reactive chlorine species on air quality.

S3. The Atmospheric Oxidative Capacity. The atmospheric oxidizing capacity (*AOC*; expressed in cm^{-3} s^{-1}), a parameter introduced by Geyer et al.[8] to account for the contribution of all oxidants, is derived here as the rate at which CO, CH_4, and NMHCs (all species are noted here as Y_i) are oxidized by the radicals of OH•, NO_3•, and Cl• as well as O_3 (noted as X_j).[8,9] Thus, when considering all combinations of the different primary pollutants and atmospheric oxidants. We write the calculation of *AOC* as below:

$$AOC = \sum_i^j k_{i,j}[Y_i][X_j].$$

Supplemental Figures

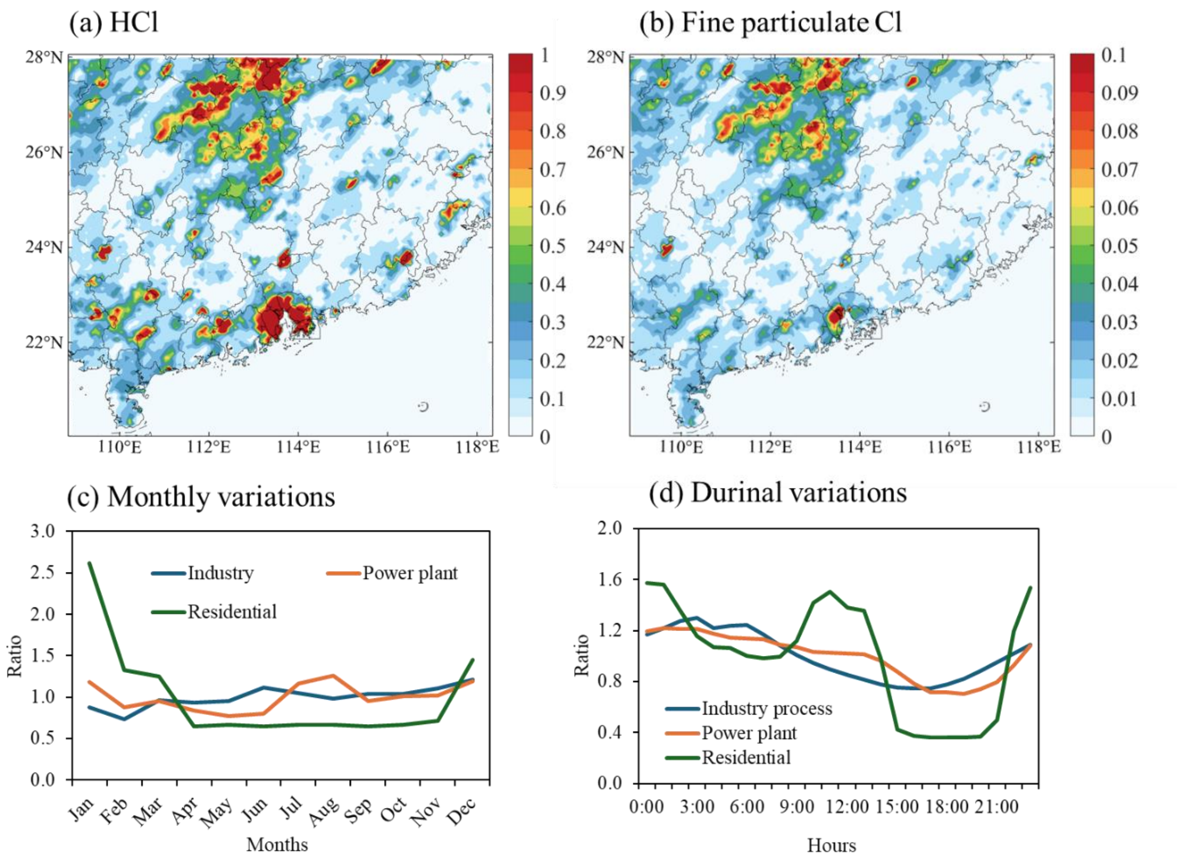

Figure S1. Spatial distribution of HCl (a, unit: mol km^{-2} hr^{-1}) and fine particulate chloride (b, unit: μg m^{-2} s^{-1}) from anthropogenic activity. The hourly (c) and monthly (d) variations in the anthropogenic chloride emissions relative to the average hourly/monthly values in different sectors.

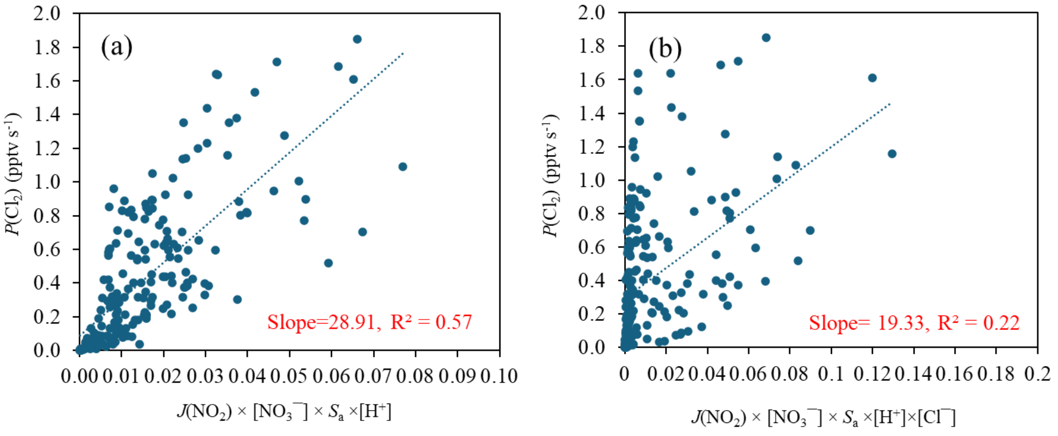

Figure S2. Relationship between the production rate of Cl_2 [$P(Cl_2)$, unit: pptv s^{-1}] and influencing factors. (a) considering $J(NO_2)$, $[NO_3^-]$, S_a, and $[H^+]$ and (b) with additional consideration of $[Cl^-]$.

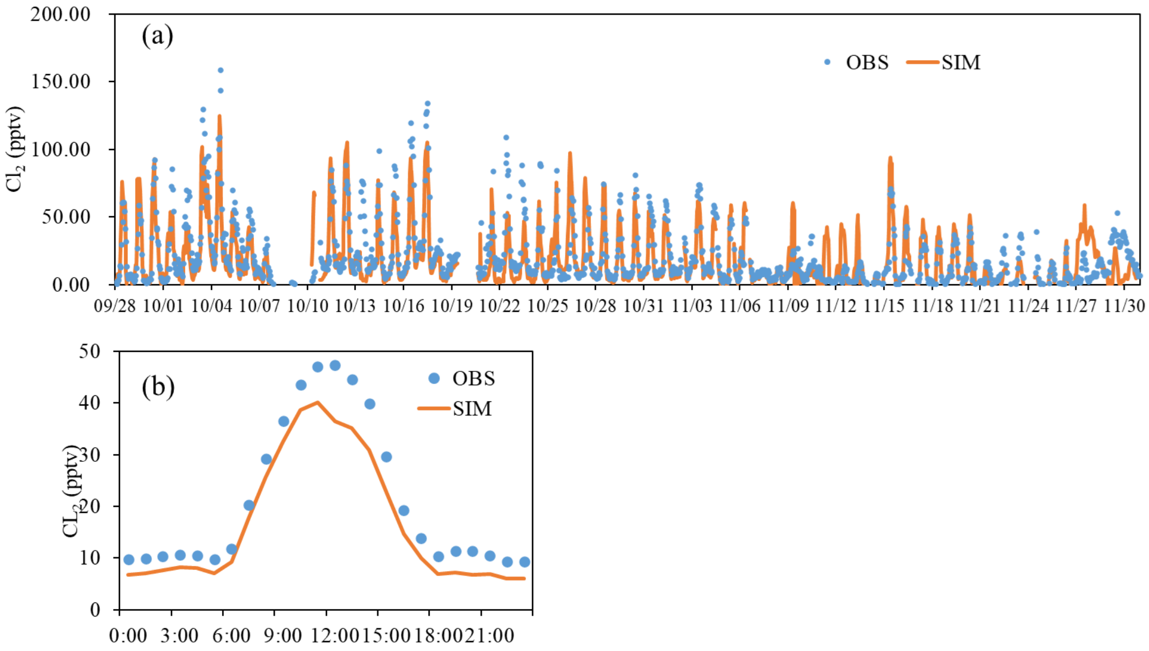

Figure S3. Model performance of Cl_2 concentration at the Cape D'Aguilar site in the autumn of 2023 (unit: pptv) (a) Hourly variations in simulated and observed mixing ratios of Cl_2. (b) Campaign-averaged diurnal variations in observed and simulated mixing ratios of Cl_2.

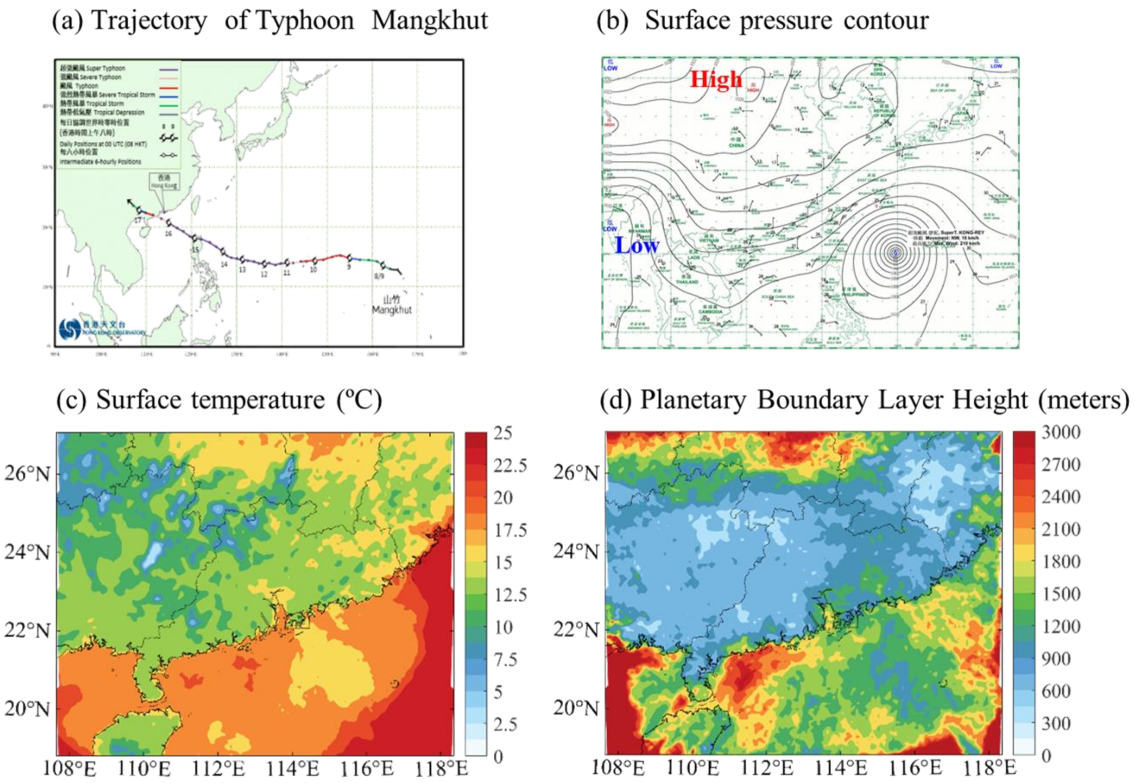

Figure S4. Meteorological conditions in field campaign period. (a) Trajectory of super typhoon "Mangkhut". (b) Contour of surface pressure in continental air (from September 4 to September 14 and from September 22 to October 7). (c-d) Spatial distribution of simulated (in CL case) surface temperature (c, unit: ºC) and planetary boundary layer height (d, unit: meters) in continental air. Panels (a-b) are obtained from the Hong Kong Observatory (https://www.hko.gov.hk/tc/).

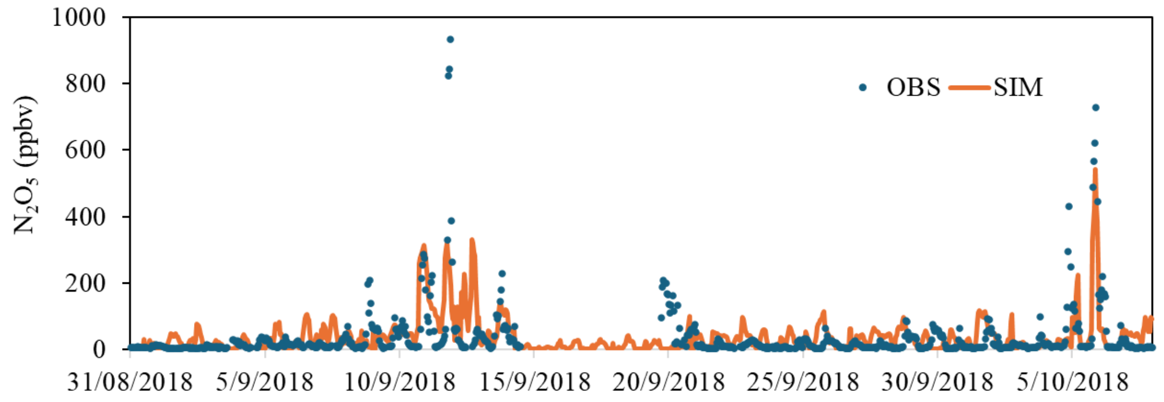

Figure S5. Hourly variations in simulated and observed mixing ratios of N_2O_5 (unit: pptv) at Cape D' Aguilar site.

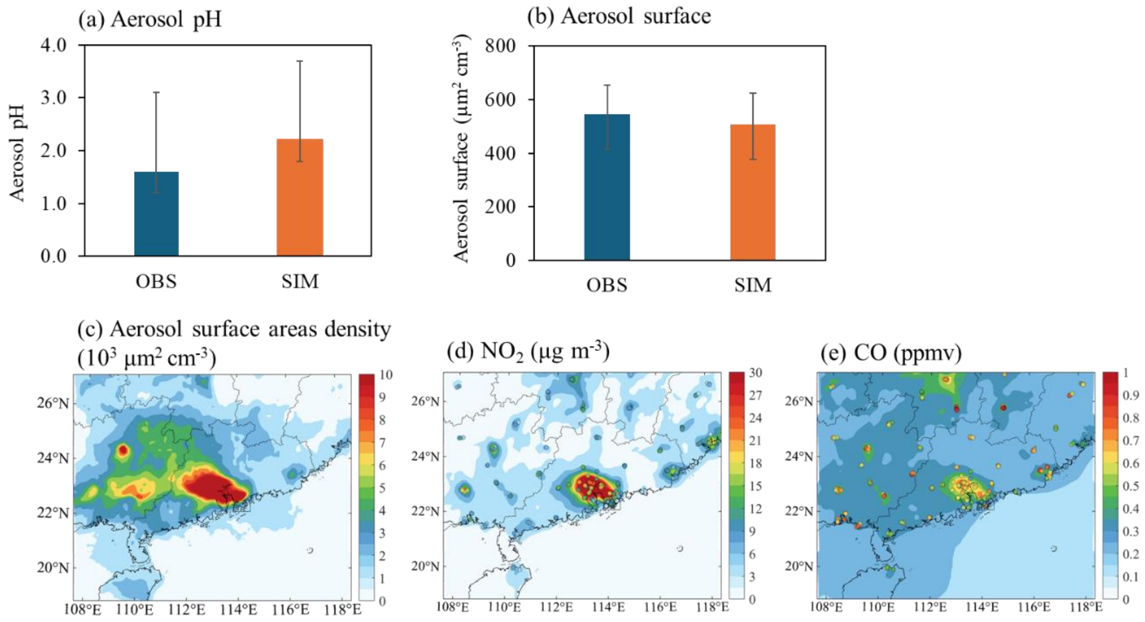

Figure S6. (a, b) Comparisons of simulated (in CL case) and observed value of (a) aerosol pH and (b) aerosol surface at Cape D' Aguilar site in continental air. The observations of aerosol pH and surface density are calculated by the off-line ISORROPIA model (see Text S1) constrained by observations. (c-e) Spatial distribution of (c) aerosol surface areas density, (d) NO_2 concentrations, and (e) CO concentrations in surface continental air in South China overplotted with available observations in South China.

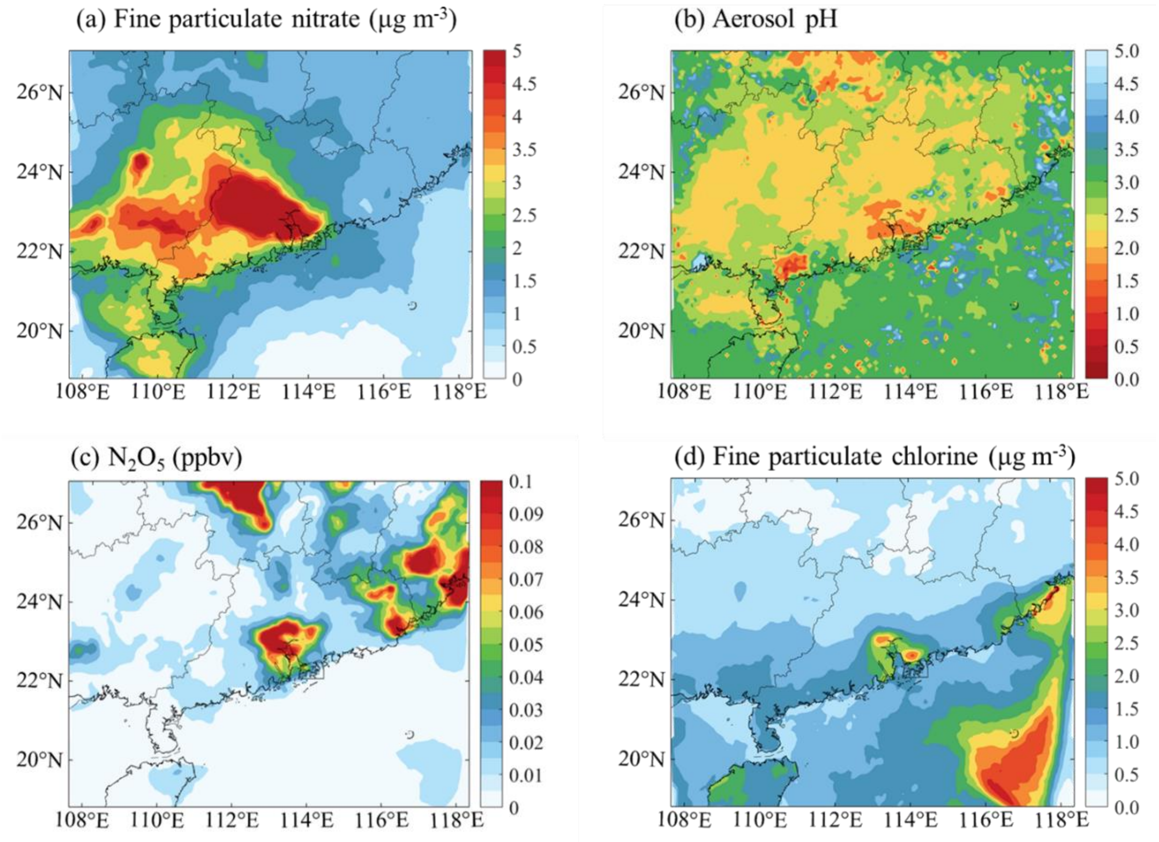

Figure S7. Spatial distribution of simulated (a) fine particulate nitrate concentration, (b) aerosol pH value, (c) N_2O_5 concentration, and (d) fine particulate chloride concentration (in CL case) in continental surface air in South China.

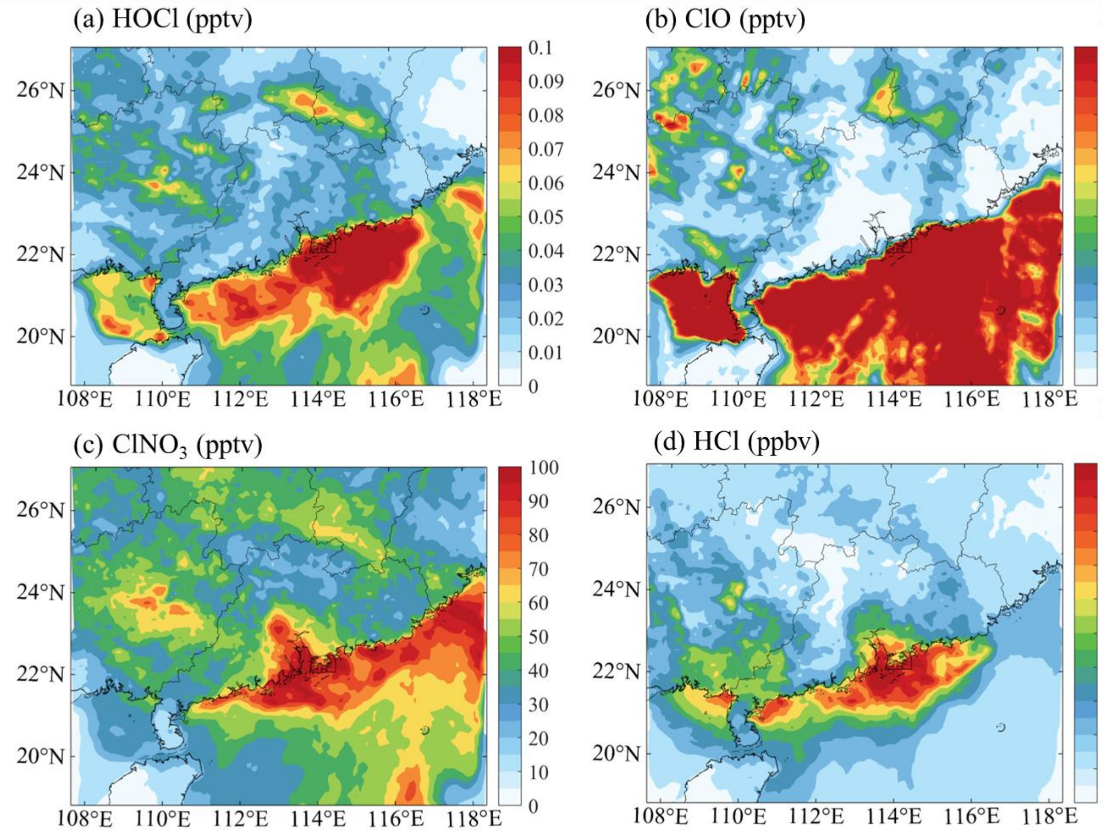

Figure S8. Spatial distribution of simulated mixing ratio of (a) HOCl, (b) ClO, (c) $ClNO_3$, and (d) HCl (in CL case) in continental surface air in South China.

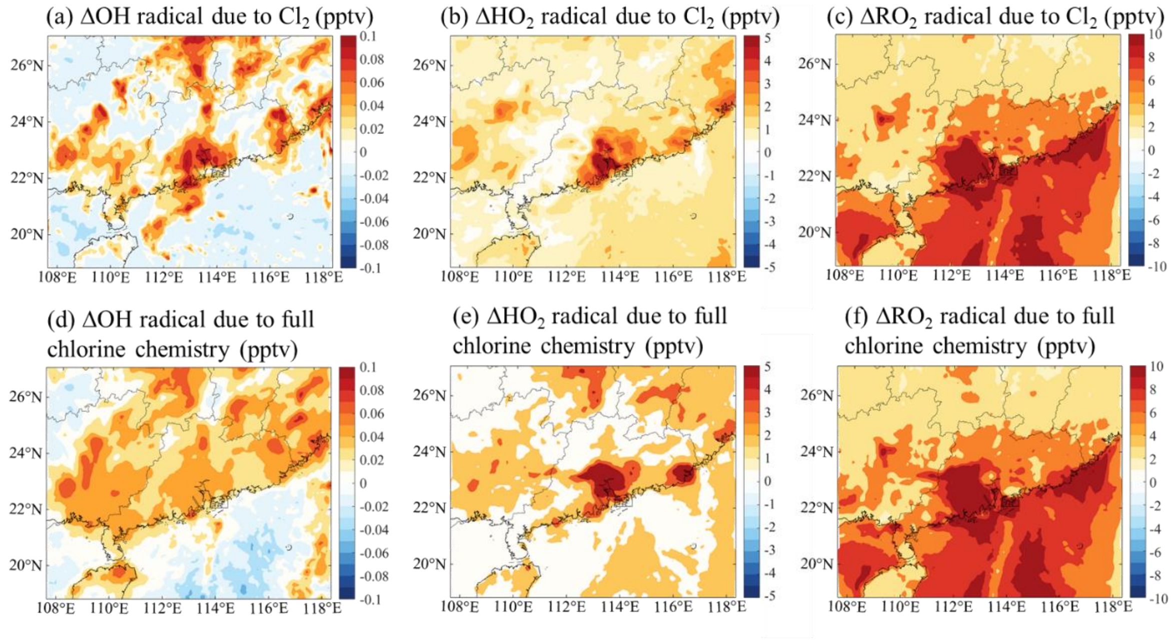

Figure S9. Changes in the mixing ratios of (a, d) daytime OH• (06:00 to 19:00 LST), (b, e) daytime HO_2•, and (c, f) RO_2• radicals in continental air due to the Cl_2 production (a-c; wCl_2 case-BASE case) and due to all chlorine-related reactions (d-f; CL case-BASE case).

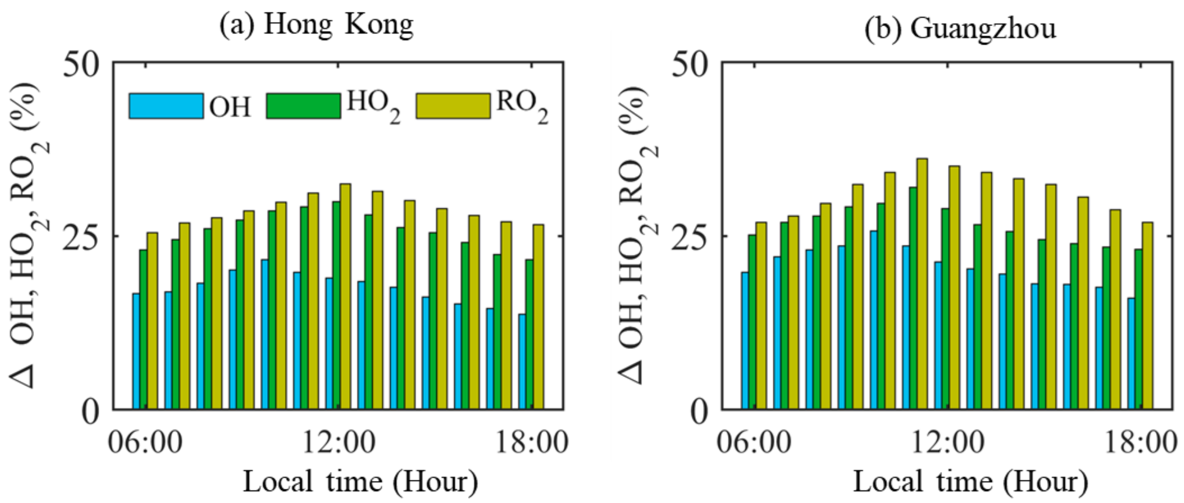

Figure S10. Percentage changes in the levels of OH•, HO_2•, and RO_2• radicals due to the Cl_2 productions (wCl_2 case-BASE case) at the monitoring sites in Hong Kong and Guangzhou in continental air during daytime (06:00 to 19:00 LST).

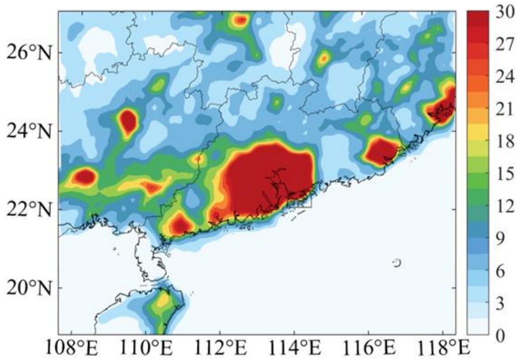

Figure S11. Spatial distribution of simulated concentration in total VOCs (unit: ppbv; in CL case) in continental surface air.

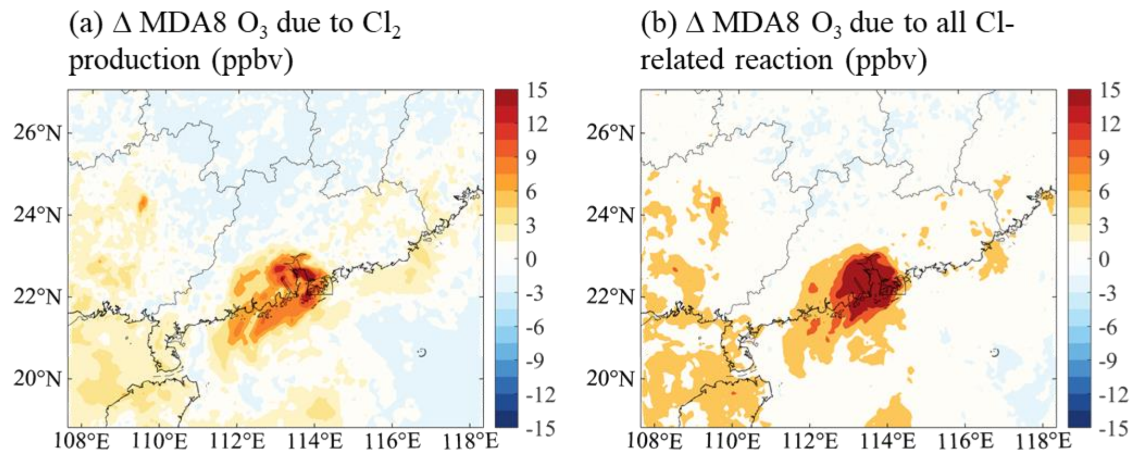

Figure S12. Changes in concentration of Maximum Daily 8-hour Average (MDA8) ozone due to (a) Cl_2 (wCl_2 case-BASE case) and (b) all chlorine-related reactions (CL case – BASE case) during continental air.

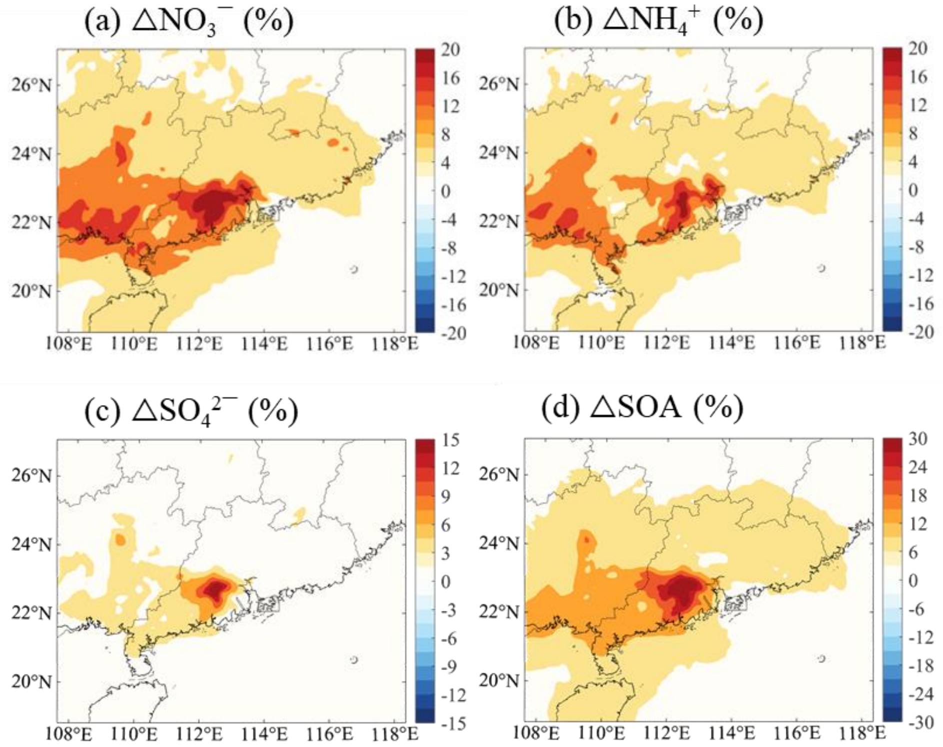

Figure S13. Percentage changes in the fine particulate (a) nitrate (NO_3^-), (b) ammonia (NH_4^+), (c) sulfate (SO_4^{2-}), and (d) secondary organic aerosols (SOA) due to Cl_2 production (wCl_2 case − BASE case) in continental air.

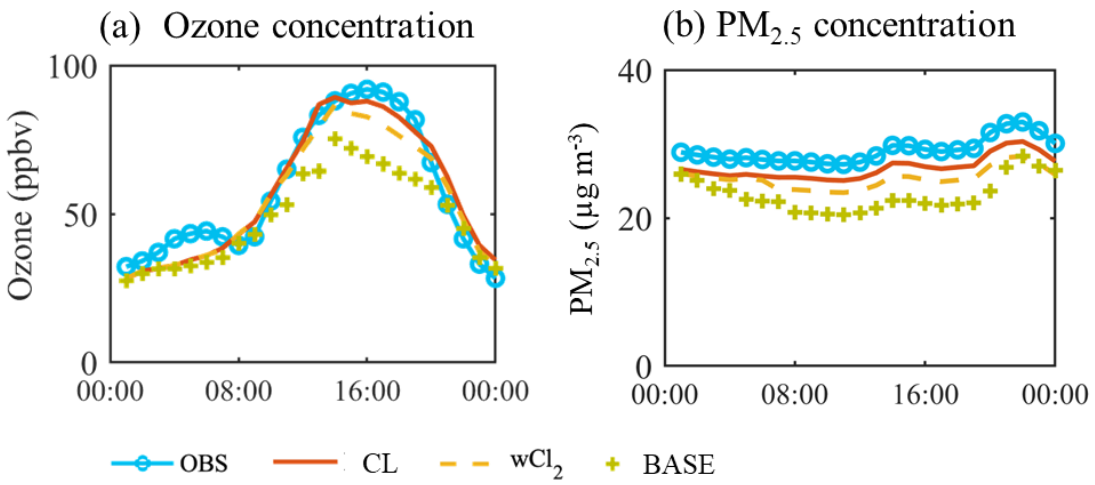

Figure S14. Diurnal variation of observed (OBS) and simulated (in the BASE, wCl_2, and CL cases) concentration of ozone and $PM_{2.5}$ at the monitoring sites in Guangzhou.

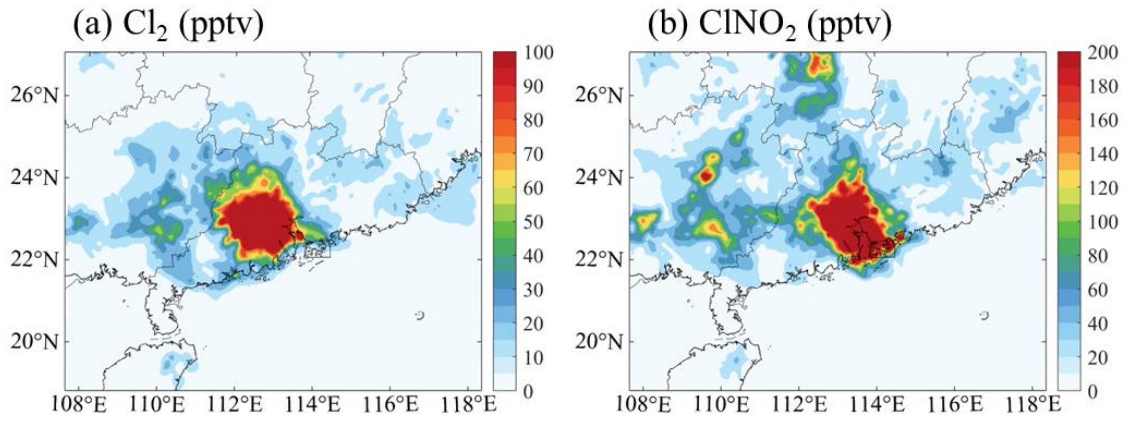

Figure S15. Spatial distribution of the mixing ratios of (a) daytime (06:00 to 19:00 LST) Cl_2 and (b) nighttime (20:00 to 05:00 LST) $ClNO_2$ with emission reduction in NO_x and SO_2 by a factor of 2 (in CL_50%EMIS case).

Supplementary Tables

Table S1. Chlorine-related reactions in this study.

	Reactions	Reaction rate	References
**Photolysis reactions**			
R1	CL2+hv=2CL	j(Pj_cl2);	Zhang et al.[10]
R2	OCLO+hv=O+CLO	j(Pj_oclo);	Zhang et al.[10]
R3	HOCL+hv=CL+OH	j(Pj_hocl);	Zhang et al.[10]
R4	CLNO2+hv=CL+NO2	j(Pj_clno2);	Zhang et al.[10]
R5	CLNO3+hv=CL+NO3	j(Pj_clno3);	Zhang et al.[10]
R6	CLNO3+hv=CLO+NO2	j(Pj_clno3b);	Zhang et al.[10]
**Gas-phase reactions**			
R7	CL+O3=CLO{+O2}	ARR3(2.8d-11, 250. _dp, TEMP); [a]	Zhang et al.[10]
R8	CL+HO2=HCL{+O2}	ARR3(1.4d-11, -270. _dp, TEMP);	Zhang et al.[10]
R9	CL+HO2=CLO+OH	ARR3(3.6d-11, 375. _dp, TEMP);	Zhang et al.[10]
R10	CL+H2O2=HCL+HO2	ARR3(1.1d-11, 980. _dp, TEMP);	Zhang et al.[10]
R11	Cl{+H2} {+O2} =HCL+HO2	ARR3(3.9d-11, 2310. _dp, TEMP);	Zhang et al.[10]
R12	Cl+NO2=ClNO2	TROE (1.8d-3, -2. d0,1.0d-10, -1. d0,0.6d0, TEMP, C_M); [b]	Zhang et al.[10]
R13	CLO+OH=Cl+HO2	ARR3(7.3d-12, 300. _dp, TEMP) ×0.94d0;	Zhang et al.[10]
R14	CLO+OH=HCl{+O2}	ARR3(7.3d-12, 300. _dp, TEMP) ×0.06d0;	Zhang et al.[10]
R15	CLO+HO2=HOCL	ARR3(2.2d-12, -340. _dp, TEMP);	Zhang et al.[10]
R16	ClO+O3=Cl{+2O2}	1.5d-17;	Zhang et al.[10]
R17	CLO+NO=CL+NO2	ARR3(6.2d-12, -295. _dp, TEMP);	Zhang et al.[10]
R18	CLO+NO2=CLNO3	TROE (1.8d-31, -3.4D0,1.5d-11, -1.9d0,0.6d0, TEMP, C_M);	Zhang et al.[10]
R19	CLO+CLO=2CL{+O2}	ARR3(3.0d-11,2450. _dp, TEMP);	Zhang et al.[10]
R20	CLO+CLO=CL2{+O2}	ARR3(1.0d-12,1590. _dp, TEMP);	Zhang et al.[10]
R21	CLO+CLO=OCLO+CL	ARR3(3.5d-13,1370. _dp, TEMP);	Zhang et al.[10]
R22	HCL+OH=H2O+CL	ARR3(1.7d-12,230. _dp, TEMP);	Zhang et al.[10]
R23	HOCL+OH=ClO+H2O	ARR3(3.0d-12,500. _dp, TEMP);	Zhang et al.[10]

R24	CL+CLNO3=CL2+NO3	ARR3(6.2d-12, -145. _dp, TEMP);	Zhang et al.[10]
R25	CLNO3+OH=0.5CLO+0.5HNO3+ 0.5HOCL+0.5NO3	ARR3(1.2d-12,330. _dp, TEMP);	Zhang et al.[10]
R26	CLNO2+OH=HOCL+NO2	ARR3(2.4d-12,1250. _dp, TEMP);	Zhang et al.[10]
R27	CL+CH4=HCL+CH3O2	ARR3(6.6d-12,1240. _dp, TEMP);	Badia et al.[11]
R28	CL+CH2O=HCL+HO2+CO	ARR3(8.1d-11,34. _dp, TEMP);	Badia et al.[11]
R29	Cl+CH3CHO=HCl+CH3CO3	8.0d-11;	Badia et al.[11]
R30	Cl+CH3OH=HCl+HO2+CH2O	5.5d-11;	Badia et al.[11]
R31	Cl+CH3OOH=HCl+CH3O2	5.7d-11;	Badia et al.[11]
R32	Cl+CH3O2=0.5CH2O+0.5CO+ 0.5H2O+0.5HO2+0.5HCl+0.5ClO	1.6d-10;	Badia et al.[11]
R33	CLO+CH3O2=CL+CH2O+HO2	ARR3(3.3d-12,115. _dp, TEMP);	Badia et al.[11]
R34	Cl+C3H8=HCl+C3H7O2	ARR3(7.85d-11,80. _dp, TEMP);	Badia et al.[11]
R35	CL+C2H6=HCL+C2H5O2	ARR3(7.2d-11,70. _dp, TEMP);	Badia et al.[11]
R36	Cl+C3H6{+O2} =HCL+PO2	3.6d-12;	Badia et al.[11]
R37	CL+BIGENE=ENEO2+HCL	2.5d-10;	This work. Based on Li et al.[12]
R38	CL+BIGALK=ALKO2+HCL	5.0d-11;	This work. Based on Li et al.[12]
R39	CL+ISOP=ISOPO2+HCL	4.3d-10;	This work. Based on Li et al.[12]
R40	CL+TOLUENE=0.18CRESOL+ 0.10TEPOMUC+0.07BZOO+ 0.65TOLO2+0.28HO2+HCL	6.1d-11;	This work. Based on Li et al. [12]
R41	CL+XYLENES=0.15XYLOL+ 0.23TEPOMUC+0.06BZOO+ 0.56XYLENO2+0.38HO2+HCL	1.2d-10;	This work. Based on Li et al.[12]
R42	CL+APIN=TERPO2+HCL	4.7d-10;	This work. Based on IUPAC.
R43	CL+BPIN=TERPO2+HCL	3.8d-10;	This work. Based on IUPAC.
R44	CL+LIMON=TERPO2+HCL	6.4d-10;	This work. Based on IUPAC.
R45	CL+MBO=MBOO2+HCL	2.2d-10;	This work. Based on IUPAC.
**Heterogeneous reactions**			
R46	N2O5+H2O+CL$^-$=HNO3+ClNO2		Dai et al.[13]
R47	$CL^- \xrightarrow{NO3-,H+} 0.5\ CL2$	$k_1[H^+]\ [NO_3^-]\ J(NO_2)\ Sa$. k1=28.91	This work.
R48	$CL^- \xrightarrow{NO3-,H+,ORG} 0.5\ CL2$	$\frac{k_2[H^+][Cl^-]}{k_2[H^+][Cl^-]+k_3[Cl^-]+[H_2O]+k_4[Org]}$; k2=19.38; k3=483; k4=2.06;	This work. Based on Xia et al.[14]
**SOA formation**			
R49	CL+BIGALK=CL+BIGALK+CVA SOA4	5.0d-11×vbs_yield_cl (nume, den, vbs_alk5, vbs_c1000); [c]	This work. Based on Li et al.[12]

R50	CL+BIGALK=CL+BIGALK+CVASOA3	5.0d-11×vbs_yield_cl (nume, den, vbs_alk5, vbs_c100); [c]	This work. Based on Li et al.[12]
R51	CL+BIGALK=CL+BIGALK+CVASOA2	5.0d-11×vbs_yield_cl (nume, den, vbs_alk5, vbs_c10); [c]	This work. Based on Li et al.[12]
R52	CL+BIGALK=CL+BIGALK+CVASOA1	5.0d-11×vbs_yield_cl (nume, den, vbs_alk5, vbs_c1); [c]	This work. Based on Li et al.[12]
R53	CL+ISOP=CL+ISOP+CVBSOA4	4.3d-10×vbs_yield_cl (nume, den, vbs_isop, vbs_c1000);	This work. Based on Li et al. [12]
R54	CL+ISOP=CL+ISOP+CVBSOA3	4.3d-10×vbs_yield_cl (nume, den, vbs_isop, vbs_c100);	This work. Based on Li et al.[12]
R55	CL+ISOP=CL+ISOP+CVBSOA2	4.3d-10×vbs_yield_cl (nume, den, vbs_isop, vbs_c10);	This work. Based on Li et al.[12]
R56	CL+ISOP=CL+ISOP+CVBSOA1	4.3d-10×vbs_yield_cl (nume, den, vbs_isop, vbs_c1);	This work. Based on Li et al. [12]
R57	CL+TOLUENE=CL+TOLUENE+CVASOA4	6.1d-11×vbs_yield_cl (nume, den, vbs_aro1, vbs_c1000);	This work. Based on Li et al.[12]
R58	CL+TOLUENE=CL+TOLUENE+CVASOA3	6.1d-11×vbs_yield_cl (nume, den, vbs_aro1, vbs_c100);	This work. Based on Li et al.[12]
R59	CL+TOLUENE=CL+TOLUENE+CVASOA2	6.1d-11×vbs_yield_cl (nume, den, vbs_aro1, vbs_c10);	This work. Based on Li et al. [12]
R60	CL+TOLUENE=CL+TOLUENE+CVASOA1	6.1d-11×vbs_yield_cl (nume, den, vbs_aro1, vbs_c1);	This work. Based on Li et al.[12]
R61	CL+APIN=CL+APIN+CVBSOA4	4.7d-10×vbs_yield_cl (nume, den, vbs_terp, vbs_c1000);	This work. Based on Li et al.[12]
R62	CL+APIN=CL+APIN+CVBSOA3	4.7d-10×vbs_yield_cl (nume, den, vbs_terp, vbs_c100);	This work. Based on Li et al. [12]
R63	CL+APIN=CL+APIN+CVBSOA2	4.7d-10×vbs_yield_cl (nume, den, vbs_terp, vbs_c10);	This work. Based on Li et al.[12]
R64	CL+APIN=CL+APIN+CVBSOA1	4.7d-10×vbs_yield_cl (nume, den, vbs_terp, vbs_c1);	This work. Based on Li et al.[12]
R65	CL+BPIN=CL+BPIN+CVBSOA4	3.8d-10×vbs_yield_cl (nume, den, vbs_terp, vbs_c1000);	This work. Based on Li et al. [12]
R66	CL+BPIN=CL+BPIN+CVBSOA3	3.8d-10×vbs_yield_cl (nume, den, vbs_terp, vbs_c100);	This work. Based on Li et al.[12]
R67	CL+BPIN=CL+BPIN+CVBSOA2	3.8d-10×vbs_yield_cl (nume, den, vbs_terp, vbs_c10);	This work. Based on Li et al.[12]
R68	CL+BPIN=CL+BPIN+CVBSOA1	3.8d-10×vbs_yield_cl (nume, den, vbs_terp, vbs_c1);	This work. Based on Li et al.[12]
R69	CL+LIMON=CL+LIMON+CVBSOA4	6.4d-10×vbs_yield_cl (nume, den, vbs_terp, vbs_c1000);	This work. Based on Li et al.[12]
R70	CL+LIMON=CL+LIMON+CVBSOA3	6.4d-10×vbs_yield_cl (nume, den, vbs_terp, vbs_c100);	This work. Based on Li et al.[12]
R71	CL+LIMON=CL+LIMON+CVBSOA2	6.4d-10×vbs_yield_cl (nume, den, vbs_terp, vbs_c10);	This work. Based on Li et al.[12]
R72	CL+LIMON=CL+LIMON+CVBSOA1	6.4d-10×vbs_yield_cl (nume, den, vbs_terp, vbs_c1);	This work. Based on Li et al.[12]

Note: **[a, b]** ARR3 and TROE function and specific kinetic data are taken from WRF-Chem v 4.1.2;[15,16] TEMP is the ambient air temperature (unit: k); C_M is the ambient air density (unit: cm^{-3}); **[c]** Calculation of SOA yield is based on Lane al.[17] and Li et al.[12]; nume and den is the reaction rate constant for the reaction of RO_2 with NO and RO_2 with HO_2, respectively; vbs_terp represents different types of VOCs; vbs_c1, vbs_c10, vbs_c100 and vbs_c1000 represent the saturation concentrations in 1, 10, 100, and 1000 (unit: μg m^{-3}) of the surrogate specie.

Table S2. HONO-related reactions in WRF-Chem model

Reactions	Reaction rate	References
$NO+OH \rightarrow HONO$	TROEMS (7.0D-31, -2.6_dp, 3.6D-11, -0.1_dp, TEMP, C_M);	Dai et al.,[6]
$NO+NO_2+H_2O \rightarrow 2HONO$	5.00×10^{-40}	Dai et al.,[6]
$HONO+HONO \rightarrow NO+NO_2+H_2O$	1.00×10^{-20}	Dai et al.,[6]
$HONO+OH \rightarrow NO_2+H_2O$	2.50×10^{-12}	Dai et al.,[6]
$NO_2 \rightarrow 0.5HONO+0.5HNO_3$	-	Dai et al.,[6]
$PNO_3^- \rightarrow 0.67HONO+0.33NO_2$	: $J_{PNO3} = (8.3\times10^{-5}/7\times 10^{-7}) \times J_{HNO3}$	Dai et al.,[25]

Table S3. Physical modules used in model simulations

Atmospheric process	Scheme
Cloud microphysics	Morrison double moment[18]
Cumulus parameterization	Grell 3D Ensemble Scheme[19]
Land-surface physics	Noah Land Surface Model[20]
Longwave radiation	RRTM scheme[21]
Shortwave radiation	RRTM scheme[21]
Planetary boundary layer	Yonsei University PBL[22]
Photolysis	Madronich Fast Tropospheric Ultraviolet-Visible (FTUV)[23,24]

Table S4. Statistical analysis of model performance for meteorological parameters and air pollutants in South China.

	SIM	OBS	Bias	R	NMB	NME
Relative humidity (%)	80.2	82.0	-1.8	0.94	-7.8%	18.9%
Wind speed (m s^{-1})	5.3	4.5	0.8	0.90	8.9%	19.2%
Surface temperature (ºC)	28.4	29.0	-0.6	0.94	-6.2%	15.2%
Ozone (μg m^{-3})	40.8	46.3	-5.5	0.86	-12.8%	24.5%
NO_2 (μg m^{-3})	32.5	29.1	3.4	0.62	5.2%	20.9%
$PM_{2.5}$ (μg m^{-3})	42.0	48.2	-6.2	0.78	-13.5%	29.2%

Note: SIM and OBS represent the average of calculated and measured value of meteorological parameters or concentrations of chemicals. Bias is the mean bias calculated as the difference between SIM and OBS; *R* is the correlation coefficient (unitless); NMB is the normalized mean bias (unit: %); NME is the normalized mean error (unit: %).

Supplementary References

(1) Fountoukis, C. and Nenes, A.: ISORROPIA II: a computationally efficient thermodynamic equilibrium model for K+–Ca2+–Mg2+–NH4+–Na+–SO42−–NO3−–Cl−–H2O aerosols, *Atmos. Chem. Phys*., 2007, 7, 4639–4659.

(2) Shen, H., Chen, Z., Li, H., Qian, X., Qin, X., Shi, W. Gas-particle partitioning of carbonyl compounds in the ambient atmosphere. *Environ. Sci. Technol,* 2018, 52(19), 10997-11006.

(3) Dai, J., Wang, X., Dai, W., Chang, M. The impact of inhomogeneous urban canopy parameters on meteorological conditions and implication for air quality in the Pearl River Delta region. *Urban Clim.,* 2019, 29, 100494.

(4) Huang, Z. J., Zhong, Z. M., Sha, Q. G., Xu, Y. Q., Zhang, Z. W., Wu, L. L., Wang, Y. Z., Zhang, L. H., Cui, X. Z., Tang, M. S., Shi, B. W., Zheng, C. Z., Li, Z., Hu, M. M., Bi, L. L., Zheng, J. Y., and Yan, M.: An updated model-ready emission inventory for Guangdong Province by incorporating big data and mapping onto multiple chemical mechanisms, *Sci. Total Environ.,* , 2021 769, 144535.

(5) Huang, X., Li, M., Li, J., Song, Y. A high-resolution emission inventory of crop burning in fields in China based on MODIS Thermal Anomalies/Fire products. *Atmos. Environ.,* 2012. 50, 9-15.

(6) Dai, J., Brasseur, G. P., Vrekoussis, M., Kanakidou, M., Qu, K., Zhang, Y., Zhang, H., and Wang, T.: The atmospheric oxidizing capacity in China – Part 1: Roles of different photochemical processes, *Atmos. Chem. Phys.,* 2023, 23, 14127–14158.

(7) Chen, Y., Cheng, Y., Ma, N., Wolke, R., Nordmann, S., Schüttauf, S., Wiedensohler, A. Sea salt emission, transport and influence on size-segregated nitrate simulation: a case study in northwestern Europe by WRF-Chem. *Atmos. Chem. Phys.,* 2016,16 (18), 12081-12097.

(8) Geyer, A., Alicke, B., Konrad, S., Schmitz, T., Stutz, J. and Platt, U.: Chemistry and oxidation capacity of the nitrate radical in the continental boundary layer near Berlin, *J. Geophys. Res. Atmos,* 2001, 106, 8013–8025.

(9) Elguindi, N., Granier, C., Stavrakou, T., Darras, S., Bauwens, M., Cao, H., et al. Intercomparison of magnitudes and trends in anthropogenic surface emissions from bottom-up inventories, top-down estimates, and emission scenarios., *Earth's Future*, 2020, 8, e2020EF001520.

(10) Zhang, L., Li, Q., Wang, T., Ahmadov, R., Zhang, Q., Li, M., and Lv, M.: Combined impacts of nitrous acid and nitryl chloride on lower-tropospheric ozone: new module development in WRF-Chem and application to China, *Atmos. Chem. Phys*., 2017, 17, 9733–9750.

(11) Badia, A.; Reeves, C. E.; Baker, A. R.; Saiz-Lopez, A.; Volkamer, R.; Koenig, T. K.; Apel, E. C.; Hornbrook, R. S.; Carpenter, L. J.; Andrews, S. J.; Sherwen, T.; von Glasow, R. Importance of reactive halogens in the tropical marine atmosphere: a regional modelling study using WRF-Chem. *Atmos. Chem. Phys.* 2019, 19 (5), 3161−3189.

(12) Li, Q.; Fu, X.; Peng, X.; Wang, W.; Badia, A.; Fernandez, R. P.; Cuevas, C. A.; Mu, Y.; Chen, J.; Jimenez, J. L.; Wang, T.; Saiz-Lopez. A. Halogens Enhance Haze Pollution in China. *Environ. Sci. Technol.*, 2021, 55 (20), 13625−13637.

(13) Dai, J., Liu, Y., Wang, P., Fu, X., Xia, M., Wang, T. The impact of sea-salt chloride on ozone through heterogeneous reaction with N2O5 in a coastal region of south China. *Atmos. Environ.,* 2020, 236, 117604.

(14) Xia, M., Peng, X., Wang, W., Yu, C., Sun, P., Li, Y., Liu, Y., Xu, Z., Wang, Z., Xu, Z., Nie, W., Ding, A., and Wang, T., Significant production of ClNO2 and possible source of Cl2 from N2O5 uptake at a suburban site in eastern China. *Atmos. Chem. Phys* , 2020, 20, 6147–6158.

(15) Skamarock, W. C., Klemp, J. B., Dudhia, J., Gill, D. O., Liu, Z., Berner, J., Wang, W., Powers, J. G., Duda, M. G., Barker, D. M., and Huang, X.-Y.: A Description of the Advanced Research WRF Model Version 4, *Tech. rep.,* UCAR/NCAR, 2019.

(16) Emmons, L. K., Walters, S., Hess, P. G., Lamarque, J.-F., Pfister, G. G., Fillmore, D., Granier, C., Guenther, A., Kinnison, D., Laepple, T., Orlando, J., Tie, X., Tyndall, G., Widmeyer, C., Baughcum, S. L., and Kloster, S.: Description and evaluation of the Model for Ozone and Related chemical Tracers, version 4 (MOZART-4), *Geosci. Model Dev.,* 2010, 3, 43–67,.

(17) Lane, T. E.; Donahue, N. M.; Pandis, S. N. Simulating secondary organic aerosol formation using the volatility basis-set approach in a chemical transport model. *Atmos. Environ.* 2008, 42(32), 7439−7451.

(18) Morrison, H. C. J. A., J. A. Curry, and V. I. Khvorostyanov. A new double-moment microphysics parameterization for application in cloud and climate models. Part I: Description., *J. Atmos. Sci.,* 2005, 62.6: 1665-1677.

(19) Grell, G. A., and Dévényi, D., A generalized approach to parameterizing convection combining ensemble and data assimilation techniques, *Geophys. Res. Lett.,* 2002, 29 (14).

(20) Chen, F., Dudhia, J. Coupling an Advanced Land Surface–Hydrology Model with the Penn State–NCAR MM5 Modeling System. Part I: Model Implementation and Sensitivity, *Monthly Weather Review*, 2001, 129(4), 569-585.

(21) Mlawer, E. J., Taubman, S. J., Brown, P. D., Iacono, M. J., and Clough, S. A. Radiative transfer for inhomogeneous atmospheres: RRTM, a validated correlated-k model for the longwave, J. Geophys. Res., 1997, 102 (D14), 16663– 16682.

(22) Hong, S., Noh, Y., and Dudhia, J. A New Vertical Diffusion Package with an Explicit Treatment of Entrainment Processes. *Monthly Weather Review*, 2006, 134, 9, 2318-2341.

(23) Madronich, S. Photodissociation in the atmosphere: 1. Actinic flux and the effects of ground reflections and clouds., *J. Geophys. Res. Atmos*, 1987, 92(D8), 9740-9752.

(24) Fast, J. D., Gustafson, W. I., Easter, R. C., Zaveri, R. A., Barnard, J. C., Chapman, E. G., Grell, G. A., and Peckham, S. E., Evolution of ozone, particulates, and aerosol direct radiative forcing in the vicinity of Houston using a fully coupled meteorology-chemistry-aerosol model,

J. Geophys. Res. Atmos, 2006, 111, D21305.

(25) Dai, J. and Wang, T.: Impact of international shipping emissions on ozone and $PM_{2.5}$ in East Asia during summer: the important role of HONO and $ClNO_2$, *Atmos. Chem. Phys.,* 2021, 21, 8747–8759.
